# Computer-Generated, Mechanistic Networks Assist in Assigning the Outcomes of Complex Multicomponent Reactions

**DOI:** 10.1021/jacs.5c02846

**Published:** 2025-04-28

**Authors:** Maciej Krzeszewski, Olena Vakuliuk, Mariusz Tasior, Agnieszka Wołos, Rafał Roszak, Karol Molga, Mohammad B. Teimouri, Bartosz A. Grzybowski, Daniel T. Gryko

**Affiliations:** † Institute of Organic Chemistry, Polish Academy of Sciences, Ul. Kasprzaka 44/52, Warsaw 01-224, Poland; ‡ Faculty of Chemistry, Kharazmi University, South Mofateh Avenue, Tehran 15719-14911, Iran; § Allchemy, Inc., 45th Street #201, Highland, Indiana 46322, United States; ∥ IBS Center for Algorithmic and Robotized Synthesis (CARS), 50, UNIST-gil, Eonyang-eup, Ulju-gun, Ulsan 689-798, South Korea; ⊥ Department of Chemistry, UNIST, 50, UNIST-gil, Eonyang-eup, Ulju-gun, Ulsan 689-798, South Korea

## Abstract

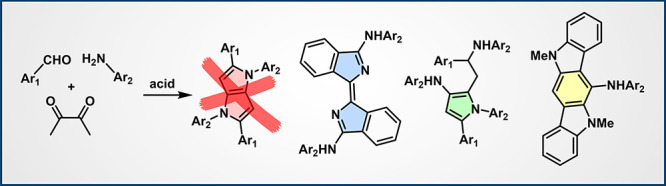

The appeal of multicomponent reactions, MCRs, is that they can offer highly convergent, atom-economical access to diverse and complex molecules. Traditionally, such MCRs have been discovered “by serendipity” or “by analogy” but recently the first examples of MCRs designed by computers became known. The current work reports a situation between these extremes whereby the MCRs were initially designed by analogy to a known class but yielded unexpected results—at which point, mechanistic-network search performed by the computer was used to aid the assignment of the majority (though not all) of experimentally obtained products. The novel MCRs we report are of interest because they (i) have markedly different outcomes for substrates differing in relatively small structural detail; (ii) offer very high increase in substrate-to-product complexity; and (iii) enable access to photoactive scaffolds with potential applications as functional dyes. In a broader context, our results highlight a productive synergy between human and computer-driven analyses in synthetic chemistry.

## Introduction

Multicomponent reactions,^1−6^ MCRs, are of lasting interest to synthetic chemists because they offer facile access to diverse scaffolds while improving step and atom economy,^7−17^ often under “green” conditions that are central to modern synthesis. MCRs have a history almost as long as synthetic chemistry itself, with Strecker’s synthesis of α-amino cyanides dating back to 1850. Subsequently, the groundbreaking contributions of Passerini and Ugi^18−20^ further propelled the development of this field, spurring discovery of over 600 classes of mechanistically and structurally diverse MCRs (catalogued and digitized in ref (21)). However, despite this surge, many MCRs are still being discovered by analogy to the already existing types (e.g., MCR synthesis of 1,2-dihydropyridine derivatives^22^ derives from the Hantzsch reaction, thioimidazolidinone formation stems from Ugi-Smiles MCR,^23^ etc.) or even by sheer serendipity. As an example of the latter, some years ago,^24−33^ we stumbled upon a novel MCR involving butane-2,3-dione, aromatic aldehydes, and aromatic primary amines, leading to various 1,4-dihydropyrrolo[3,2-*b*]pyrroles in good yields and at >10 g scales.^25^ Now, we have returned to this MCR with an initial objective to use diverse aromatic aldehydes and amines to synthesize additional derivatives for specific applications as functional dyes. Surprisingly, however, relatively small changes in the substrates’ structure resulted in qualitative changes in the reaction outcomes—the isolated products still exhibited interesting photophysical properties but did not feature the original 1,4-dihydropyrrolo[3,2-*b*]pyrrole, DHPP, scaffold. This outcome prompted an effort that combined structure-characterization experiments with computational analyses of large networks of mechanistic steps and chemically compatible step-sequences within these networks. Ultimately, this two-pronged approach allowed us to elucidate the structures of the products as well as the plausible mechanisms by which they form.

These results add to the growing list of applications of computers in nontrivial synthesis-oriented problems: from complex retrosynthetic analyses,^34^ to circular chemistry,^35^ to catalyst design^36−39^ and reaction optimization,^40,41^ to the discovery of new synthetic strategies,^42,43^ elucidation of complex reaction mechanisms^44^ and the discovery of mechanistically unprecedented MCRs.^21^ The current work establishes computational analysis of mechanistic networks as a technique auxiliary to traditional spectroscopic techniques—generating ideas as to the identity of “unexpected” reaction outcomes and guiding (or accelerating) structural assignments that, by spectroscopy alone, may be challenging or even ambiguous.

## Results and Discussion

### Reaction Selection and Outcomes

**Figure 1:**
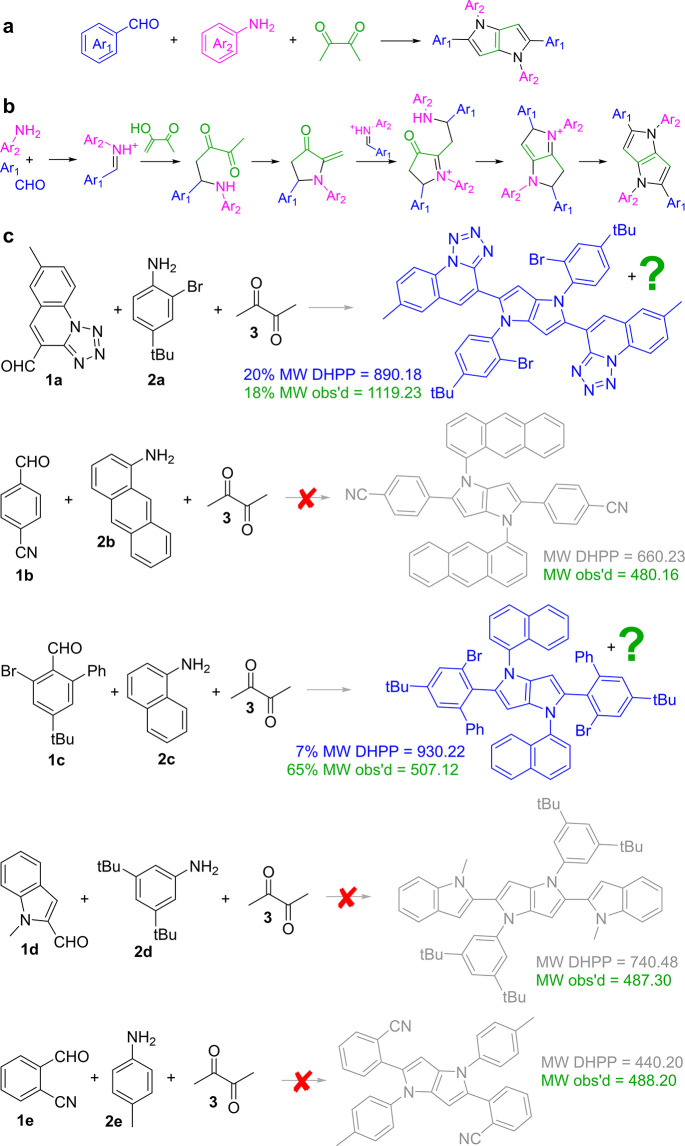
(a) A general scheme of the MCR from refs (24–30) and (b) its plausible mechanism (detailed Allchemy screenshot of this route is provided in the Supporting Information, Figure S8). (c) Specific substrates used in this study. Reactions **1x/2x/3** where **x** = **b**,**d**,**e** gave no 1,4-dihydropyrrolo[3,2-*b*]pyrrole, DHPP, scaffolds but some unknown, fluorescent compounds. Reactions **x** = **a**,**c** gave both the DHPP and some unknown fluorescent products. Each entry provides the molecular mass for the originally intended 1,4-dihydropyrrolo[3,2-*b*]pyrroles, MW DHPP, and the masses observed for the unknown species, MW obs’d. For structural assignments and yields, see Figures 2, 3 and 5.

The current work has its origins in our 2013 study,^24^ in which we showed that an aromatic aldehyde, an aromatic amine and butane-2,3-dione can engage into a mechanistic sequence of double Mannich reaction, imine formation, tautomerization and oxidation, ultimately producing a 1,4-dihydropyrrolo[3,2-*b*]pyrrole, DHPP, scaffold (Figure 1a,b). Notably, condensation of two molecules of an aromatic aldehyde with two molecules of an aromatic amine and one molecule of butane-2,3-dione is one of the very few known five-component reactions. This reaction proved easy to up-scale^25^ and versatile, leading to a range of DHPP derivatives exhibiting interesting photophysical properties (e.g., substituent-tunable Stokes shifts, large fluorescence quantum yields, large two-photon absorption cross-section).^26,27^ Owing to their centrosymmetric, quadrupolar structures, DHPPs turned out to be an excellent test bed to study symmetry breaking in the excited state,^28,29^ substantiating a new theoretical model of this phenomenon for quadrupolar dyes.^30^ In parallel, photophysical investigation of DHPPs led to the formulation of rules governing the fluorescence of nitroaromatics.^31^ It was also shown that these scaffolds constitute convenient building blocks in the construction of N-doped nanographenes.^32,33^

At the same time, among ca. 200 substrate combinations studied to date, we have catalogued five in which no DHPP products were observed or were accompanied by significant amounts of other photoactive products. These exceptions correspond to aromatic aldehydes **1a**–**e** and amines **2a**–**e** in Figure 1c. All reactions also used butane-2,3-dione (**3**) and, as in ref (25) were conducted under mildly acidic conditions, with iron(III) perchlorate for **x** = **a**,**b**,**c**, NbCl_5_ for **e** and TsOH for **d**(27) as catalysts. In all cases, conversion was observed, and the reactions gave intensely colored, unknown products. Most of these products exhibited interesting photophysical properties making them suitable candidates for functional dyes (Table 1). For instance, the product of reaction involving the **1e**/**2e** substrate pair emitted green light with λ_em_ = 522 nm and reasonably high fluorescence, Φ_fl_ = 0.51. By contrast, products of reactions involving **1a**/**2a**, **1b**/**2b**, and **1d**/**2d** substrates all emitted blue light (emission maxima between 437 to 487 nm) and had Φ_fl_ values between 0.40 and 0.48. Of note, all compounds displayed relatively low Stokes’ shift values, with the product of the **1b**/**2b** reaction having the lowest value (Δ*S* = 450 cm^–1^). This feature indicates the rigid nature of the fluorophores and suggests minimal differences between the excited S_1_ state and the ground S_0_ state geometries across the compounds.

**Table 1:** Photophysical Properties of the Non-DHPP Products of MCRs From Figure 1 (between aldehydes **1a**–**e**, amines **2a**–**e**, and dione **3**)^a^

reaction	λ_abs_ [nm]	ε·10^–3^ [M^–1^ cm^–1^]	λ_em_ [nm]	Stokes shift [cm^–1^]	Φ_fl_^b^
**1a/2a/3**	449	9	487	950	0.40
**1b/2b/3**	445	17	454	450	0.48
**1c/2c/3**	375	0.3	---	---	<0.001
**1d/2d/3**	399, 420	2.5	437	1750	0.44
**1e/2e/3**	501	25	522	800	0.51

a Measured in toluene.

b Determined with coumarin 153 in EtOH as a standard (Φ_fl_ = 0.53).

However, these products were not what we had anticipated for our “generic” MCR from Figure 1a,b. In particular, reactions **1x**/**2x**/**3** (where **x** = **b,d,e**) gave not even trace amounts of the DHPPs whereas reactions of **1a/2a/3** and **1c**/**2c**/**3** gave a mixture of DHPP and some other, fluorescent products.

To assign these products, we proceeded in two ways: (1) We embarked on a traditional characterization campaign (^1^H NMR, ^13^C NMR, ^1^H^13^C HMBC, ^1^H^13^C HSQC, HRMS). To avoid unequivocal assignments—especially for reactions **1a**/**2a**/**3** and **1e/2e/3** for which spectral analyses were particularly challenging—we also screened conditions for obtaining single crystals suitable for X-ray analyses. (2) In parallel, we performed mechanistic-level computational analyses of the mechanistic steps, in which the components of the reaction mixtures could engage.

### Mechanistic Network Analyses

We first discuss the computational analyses relying on the MECH module of our synthesis-planning Allchemy platform (available for testing by academic users at https://mech.allchemy.net). As described in detail in our recent publication^21^ on screening substrate combinations that can give rise to new MCRs, this specific algorithm uses c.a. 9000 expert-coded mechanistic transforms operating, roughly, at the level of “arrow-pushing” steps and generalized beyond specific literature precedents. These transforms are coded to take into account not only typical conditions (as in our prior works on computer-assisted synthesis^21,34,35,42−44^) but also byproducts and rudimentary information about typical speeds (categorized as very slow, slow, fast and very fast).

**Figure 2:**
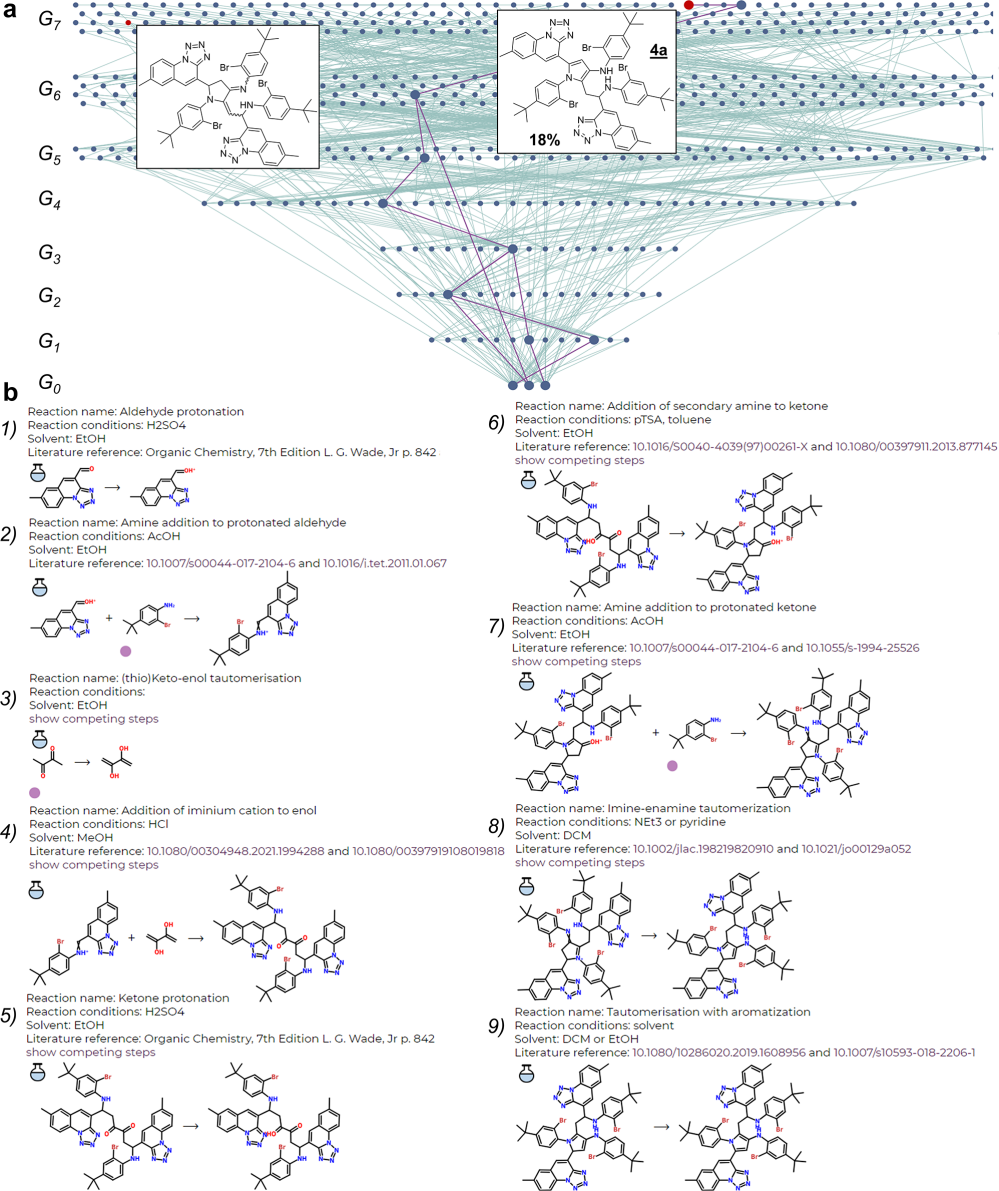
(a) Screenshot of Allchemy’s mechanistic network commencing from substrates **1a**/**2a**/**3** (three nodes in the bottom row, the “zeroth synthetic generation”, *G*_0_). The node colored red (in synthetic generation *G*_7_) is one of two products matching the experimentally recorded mass. Its structure, **4a**, is shown next to the node. Another structure with matching mass is also shown—as seen, it is the less stable tautomer of the red-node product. Violet line traces the shortest mechanistic pathway connecting this molecule to the substrates in *G*_0_. Details of the pathway are provided in the screenshots in (b). Each step also suggests typical conditions for a given type of transformation (i.e., typical but not necessarily optimal for the specific molecule to which this transform is applied). Hyperlinks direct the user to publications in which similar mechanistic transformations were discussed (again, not for the specific substrates shown here as the algorithm is based on generalized mechanistic transforms rather than specific literature precedents). Flask icons can be clicked to expand the transforms and show byproducts formed in a given reaction. Product **4a** was obtained in 18% isolated yield.

The mechanistic transforms are used to propagate mechanistic networks. They are first applied to the user-specified substrates (“synthetic generation” marked *G*_0_ in the bottom row of the network shown in Figure 2a) to give products in generation *G*_1_; then, the *G*_0_ and *G*_1_ species are combined and transforms are applied to them to generate *G*_2_ products. This procedure is iterated until the desired number *n* of generations is reached (here, up to *n* = 9). As we quantified in ref (44) on the computational prediction of carbocationic rearrangements, such mechanistic networks are densely connected and rapidly expanding with *n* (often, faster than exponentially).

Once the network is generated, the algorithm traces mechanistic sequences to all molecules/nodes in the network, removing those for which individual steps are not mutually compatible (water sensitive and water tolerant, requiring reductive and oxidative conditions, etc.). Ultimately, sequences that can work “in one flask” are retained. Assuming a sequence survives this basic scrutiny, side reactions are analyzed to make sure that they are not faster than those along the parent routes. Also, side and byproducts are allowed to react with each other. The algorithm then checks if any of the molecules in these side-networks can react with molecules in the main sequence—if so, warnings to the user are issued. The code for all these analyses is deposited at https://zenodo.org/records/13627263 and is further described in ref (21).

The mechanistic network thus constructed can be queried in various ways (e.g., to select products featuring literature-unknown scaffolds, or those offering maximal complexity increase with respect to the starting materials).

In our current work, we are not using MECH to find new, MCR-compatible substrate combinations but, instead, for given sets of substrates, to interrogate the mechanistic networks for the presence of products based on MS-recorded masses. We hypothesize that if the matching masses are found within the network, they are likely to correspond to the experimentally observed products. To assist this analysis, the key option of the software is to read in specific molecular masses and mark any matching nodes on the mechanistic network (see User Manual in the Supporting Information, to ref (21)).

This is illustrated in the aforementioned Figure 2a where the mechanistic network was propagated from substrates **1a**,^45^**2a** and **3** and, as we recall from Figure 1c, led to some unknown, non-DHPP product with molecular mass of 1119.23. Within 11 min of calculation on a server with four AMD Opteron 6380 CPUs (64 cores in total), the algorithm generated a network of 637 molecule nodes (details of setting up this and other network analyses are provided in the Supporting Information, Section S1). Only two molecules in this network matched the desired mass—the polysubstituted pyrrole product **4a** indicated by the red node and its less stable tautomer (see structures overlaid over the network). Upon clicking on the red node, the algorithm traced the shortest mechanistic pathway that connects the substrates to this product (for some longer paths, differing mostly in the ordering of steps, see Supporting Information, Section S2). This pathway is detailed in the screenshots in Figure 2b and entails protonation of the aldehyde, formation of an iminium cation, and double Mannich-type addition to butane-2,3-dione. Note that all these steps are predicted to proceed under mutually compatible, mildly acidic conditions and without reactivity conflicts—in other words, the algorithm tells us that these steps can form a viable MCR sequence. This pathway diverges from the DHPP sequence at the intermolecular—rather than intramolecular—formation of the imine intermediate in step 7. This can be reasonably attributed to the intermediate being sterically crowded, impeding intramolecular amine attack on the ketone.

**Figure 3:**
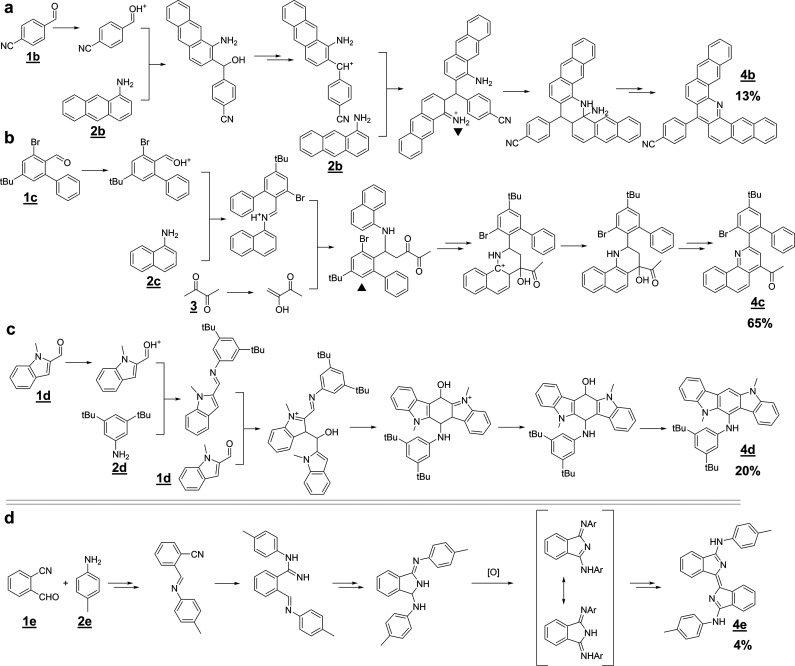
Summary of additional MCR mechanisms and products (experimentally validated) proposed (a–c) by the MECH algorithm and (d) by human chemists. Detailed Allchemy screenshots for pathways (a–c) are provided in Supporting Information, Section S2. Pathway in (a) begins with the addition of aniline to ketone, formation of a secondary carbocation (stabilized by the flanking aromatic moieties), reaction of this carbocation with the arene (within the amine substrate), cyclization via amine-to-imine addition, aromatization to acridane and final oxidation to dinaphtho[2,3-*c*:2′,3′-*h*]acridin. This product was isolated in 13% yield. (b) This sequence commences with protonation of an aldehyde and formation of an iminium cation followed by Mannich-type addition to butane-2,3-dione, protonation of ketone and intramolecular addition to naphthalene and aromatization. Sequence ends with elimination of tertiary alcohol followed by oxidation to benzoquinoline. The product is isolated in 65% yield along with 7% of the DHPP scaffold. (c) Mechanistic sequence starts with protonation of an aldehyde and formation of an iminium cation followed by addition of the second equivalent of aldehyde to indole (at 3-position), tautomerization and intramolecular addition of the imine to the second indole ring. The last step of the sequence is water elimination with aromatization forming indolo[3,2-*b*]carbazole ring system in 20% yield (when substrate **3** was also present, the yield was 2%). (d) This mechanism was not found by the algorithm but was proposed by the authors. The sequence starts from the formation of iminoamidine followed by cyclization into a five-membered ring. This cyclic aminal is oxidized to give a known^49^ diiminoisoindoline. Subsequent dimerization and elimination^48^ of toluidine gives the observed product in 4% yield.

Similar analyses were performed for the other four triples of substrates, generating networks of 600–2000 nodes on the time scales of 10–20 min each (on the same 64-core machine). In three out of four cases, the networks contained unique nodes corresponding to experimentally observed masses, and provided unique mechanistic pathways leading to these nodes. These pathways are summarized in Figure 3 with accompanying Allchemy screenshots of individual mechanistic steps shown in the Supporting Information, Section S2. The pathway in Figure 3a diverges from the DHPP sequence (Figure 1a,b) at the first step, when 1-aminoanthracene (**2b**) directly attacks the protonated aldehyde with its *ortho* carbon (instead of forming the expected iminium cation). The stabilized carbocation derived from the obtained diphenylcarbinol is subsequently attacked by the ortho carbon of **2b** to give the dearomatized iminium cation marked as ▼. The sequence is completed by intramolecular amination of ▼ and elimination to yield acridine **4b**.

In Figure 3b, a similar (but having a smaller fused ring system) aminonaphthalene substrate **2c** is used in place of **2b**—this time, the pathway leading to the product of experimentally observed mass overlaps with DHPP until the Mannich base, marked as ▲, is formed. At this step, cyclization into a six-membered ring takes place between the diketone and the ortho carbon rather than the amine from the aminonaphthalene part of ▲. Subsequent elimination and oxidation yields the benzo[*h*]quinoline **4c**. One has to note that an analogous reaction between aromatic aldehydes, aromatic amines and pyruvic acid (instead of butane-2,3-dione we used) leading to corresponding quinolines was described by Doebner already in 1887.^46^

Finally, in Figure 3c 2-formyl-3-methylindole (**1d**) is used, and the algorithm-suggested pathway diverges from the DHPP one after the formation of an imine. This imine is not attacked by the butane-2,3-dione (to give the Mannich base) but is C-3 alkylated after attacking the carbonyl group of **1d**. Interestingly, the obtained intermediate is also C-3 alkylated with the remaining imine to give pentacyclic alcohol, eliminated to indolocarbazole product **4d**.

For one reaction, involving substrates **1e**, **2e**, **3**, the network did not contain a product with the experimentally observed mass—we will discuss this case later in the text.

We note that the differences between the MECH-proposed mechanisms and the DHPP MCR from Figure 1a,b can be reasonably attributed to three properties of the substrates (1) the presence of reactive functional groups at positions ortho to the aldehyde; (2) electron-rich character of the aromatic scaffold in primary aromatic amines; (3) steric hindrance around NH_2_ or CHO groups. These factors also help rationalize why reactions **1b**/**2b**/**3** and **1d**/**2d**/**3** might not involve butane-2,3-dione (albeit these reactions are still MCRs as they involve two amines or two aldehydes).

### Experimental Product Assignments

In the absence of any algorithmic suggestions as to the identity of the experimentally observed, non-DHPP products, spectral assignments (^1^H NMR, ^13^C NMR, ^1^H^13^C HMBC, ^1^H^13^C HSQC, and HRMS) proved challenging. This is not unexpected, given the multicomponent nature of these reactions and a multitude of ways in which the signals can potentially be assigned. In contrast, with the algorithm’s suggestions in place, it is much easier to check if the shifts and coupling constants expected for these putative structures match the experimental spectra.

The unguided assignments (i.e., without any computational clues) were particularly difficult for reaction **1a**/**2a**/**3** from Figure 2 but were subsequently found congruent with the product **4a** the algorithm suggested. This product was isolated in 18% yield along with 20% of the DHPP derivative. Moreover, seeking an unequivocal proof, we later obtained crystals of quality suitable for single-crystal X-ray analysis—rewardingly, the X-ray structure shown in Figure 5c agreed with the algorithm-suggested product.

**Figure 4:**
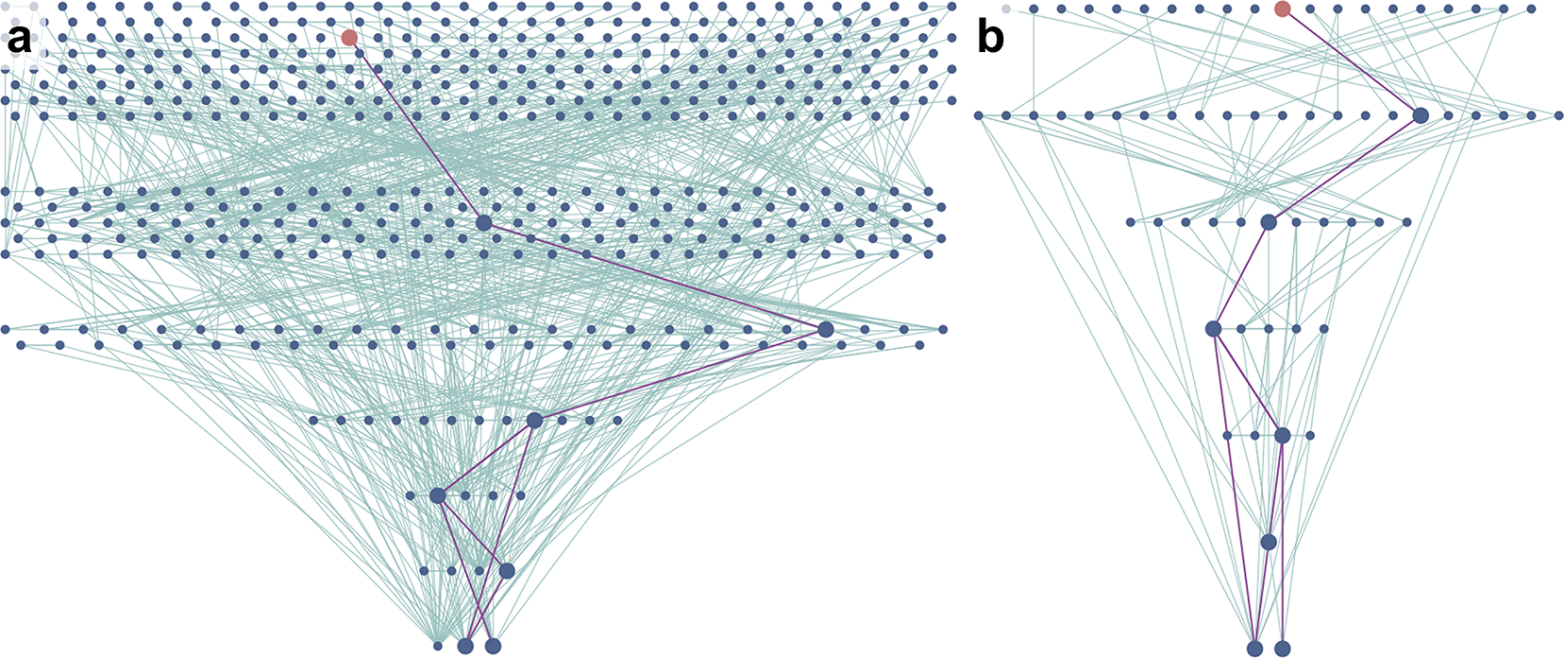
Miniatures of mechanistic networks leading to product **4d**. The larger network on the left is for the case of all three substrates, **1d/2d/3**, present. When only **1d** and **2d** substrates are used, the network (on the right) is much smaller and so is the number of competing pathways—in effect, the yield of **4d** is significantly higher (20% vs 2%). Both calculations were performed under the same class of conditions imitating the experiment (acidic conditions, high temperature; for details, see Supporting Information, Section S1).

For the three products from Figure 3a–c, NMR analyses in combination with computational predictions were sufficient to confirm the mass-matching structures found by the algorithm (for assignment details, see Supporting Information, Section S3.3). For reaction **1b/2b/3**, the sole fluorescent product shown in Figure 3a was isolated in 13% yield. Reaction **1c/2c/3** gave the product in Figure 3b in 65% yield and the DHPP one in 7% yield. For reaction **1d/2d/3**, we initially isolated only 2% of the fluorescent product shown in Figure 3c. However, guided by the algorithm’s prediction that this reaction involves two copies of **1d** and one copy of **2d** but no **3**, we repeated it with only these two substrates. This time, the isolated yield was 20% as the lack of **3** eliminated potential side reactions such as Mannich or imine formation with ketone. In Figure 4, this difference is reflected in the sizes of mechanistic networks in the presence of **3** (large network with many competing pathways) and in its absence (much smaller network with less reactions competing with a pathway leading to **4d**).

**Figure 5:**
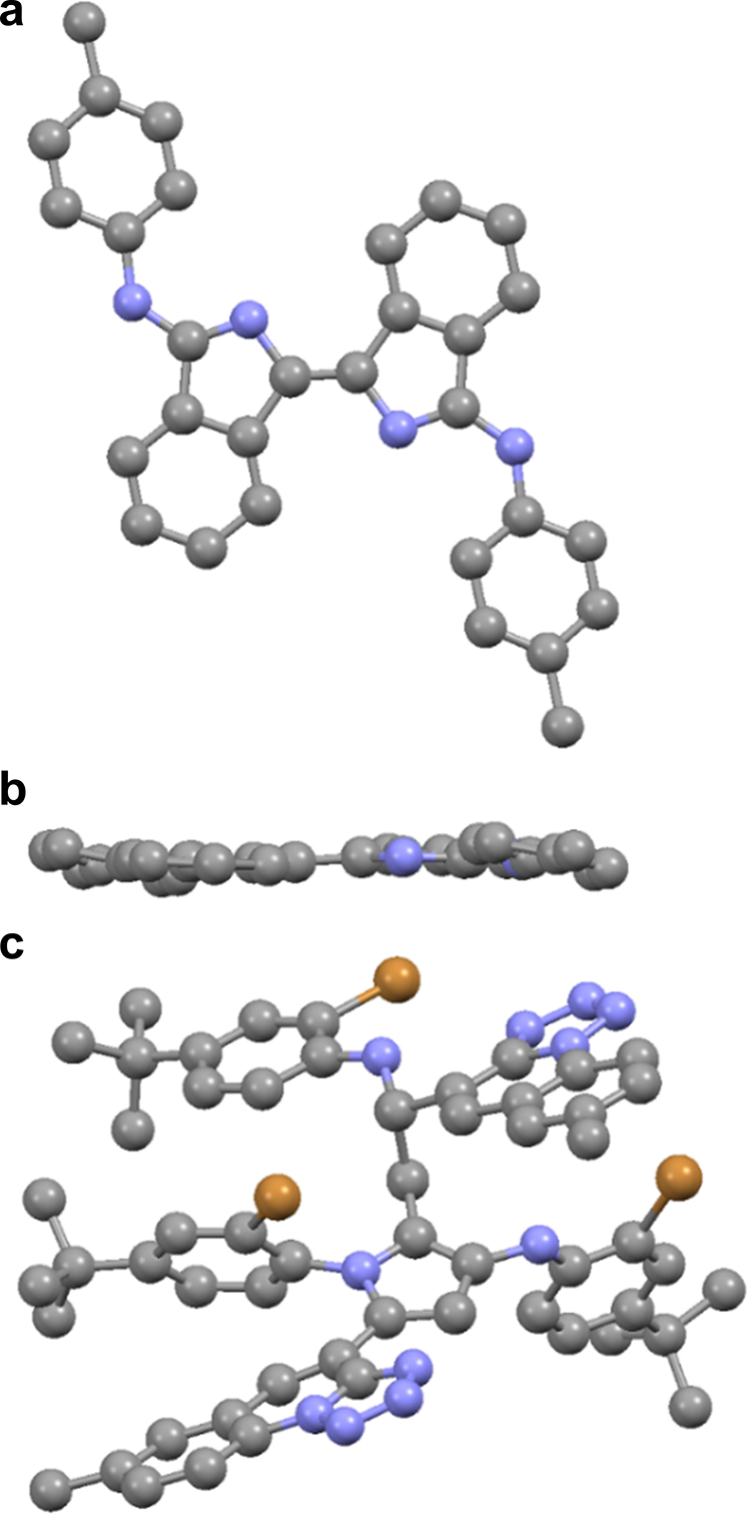
X-ray structures of products from reaction (a,b) **1e**/**2e**/**3** and (c) **1a**/**2a**/**3**. Hydrogen atoms are omitted for clarity.

For the fifth reaction, **1e/2e/3**, the one for which the algorithm did not identify any matching mass, the sole fluorescent product was isolated in only 4% yield and was determined by a combination of NMR analyses and crystallographic studies. This product, **4e**, and the putative (human-proposed) mechanism leading to it are shown in Figure 3d. The X-ray structure is shown in Figure 5a,b.

### Limitations

The “unsolved” **1e/2e/3** reaction prompts a short discussion of the algorithm’s limitations. Even though aerobic oxidation of aminals is known (e.g., for the synthesis of quinazolinones^47^), it has never been reported for uncommon five membered *N*-iminoaminals—hence, our algorithm was unaware of these key mechanistic steps and was therefore not able to predict the reaction’s product. Naturally, the missing mechanistic transform can be easily added to the algorithm’s knowledge base but this particular example is corner-case given a very poor, only 4% yield of the MCR in question. If coded too promiscuously, with a narrow reaction “core,” such a transform could lead to many false-positive predictions for other substrates, in reality offering only marginal yields. Additionally, dimerization-elimination of diiminoisoindoline was reported in only one example in Elvidge’s 1956 study^48^—this rule can be coded verbatim for only this example, but allowing for its broader use would be prudent only if more experimental information about the scope becomes available.

Another potential limitation is that for a given set of substrates, more than one product of a given mass may be identified—here, we only saw two tautomers (in Figure 2) but, in general, there may be multiple different structures formed by altogether different mechanisms. In this respect, the algorithm should be considered as a tool providing structural and mechanistic suggestions (to be cross-checked against experimental spectra) as opposed to definitive answers.

More broadly, the MECH algorithm is a work in progress and, either for the de novo design of new MCRs (as in ref (21)) or for the assignment of experimental reaction outcomes (as in here), requires numerous further improvements. Although the algorithm has been “taught” some 9000 mechanistic transforms, this knowledge base is still not complete. For example, MECH currently knows mechanisms of only basic organometallic reactions and does not yet incorporate any radical-based steps whose addition would be very timely yet is nontrivial in terms of generalization (much like for carbocationic rearrangement steps, for which additional and specialized heuristics were needed to properly define these transforms’ scope, see ref (44)). In addition, mechanistic transforms take into account steric and electronic requirements near the reaction center but are unaware of the 3D structures of entire molecules.^50^ This is potentially limiting when molecules become large/complex and their conformations dictate steric accessibility of the reacting species. Therefore, it would be desirable for the future extensions of MECH to take into account conformational analysis, although it should be remembered that such analyses would drastically increase the calculation times. Finally, calculation of kinetic barriers—which is prerequisite to estimating the yields of various species present in the mechanistic networks—remains a challenge, with a trade-off between calculation times and accuracy (as detailed in ref (44)) posing a question as to the computing power needed to carry out such analyses.

## Conclusions

To conclude, we have discovered by serendipity and explained with the help of a computer several novel MCRs—diverse both in terms of mechanisms and in terms of products’ structures (e.g., π-extended acridine, benzo[*h*]quinoline or densely substituted pyrrole derivative). In four out of five cases, the computer was extremely helpful in guiding product assignments. Since these computational analyses took only minutes (as opposed to days or even weeks for the spectroscopic and crystallization efforts), we suggest that mechanism-oriented algorithms like the one we described can be very helpful in accelerating synthesis-oriented discovery, at least at the level of hypothesis generation.

While here and in refs (21,44) we focused on forward-propagation of mechanistic networks (from substrates to products), we also see exciting opportunities to apply them in retrosynthetic direction. We envision that such analyses could suggest unprecedented means of accessing complex scaffolds, beyond the approaches based on the already known methodologies.

## Data Availability

The authors have cited additional references within the Supporting Information S1–S7. Codes for network expansion and MCR analysis are deposited at https://zenodo.org/records/13381201. The WebApp of Allchemy’s MECH module is available for testing by academic users at https://mech.allchemy.net (given the server capacity, for 10 concurrent users in two-weeks slots). For security reasons, new accounts need to be registered by sending an e-mail to admin@allchemy.net from your academic address.

## Supporting Information Available

Supporting Information provides additional theoretical and experimental details as well as spectral data (PDF)

## Abbreviations

MCR: multicomponent reaction
TsOH: *p*-toluenesulfonic acid
EtOH: ethanol
DHPP: 1,4-dihydropyrrolo[3,2-*b*]pyrrole
