# Supplementary material for: Computer-Generated, Mechanistic Networks Assist in Assigning the Outcomes of Complex Multicomponent Reactions
Source: J Am Chem Soc. 2025 Apr 28;147(18):15636–44. doi: 10.1021/jacs.5c02846 (PMC12063162; doi:10.1021/jacs.5c02846)
Supplement: Supplementary file 1 — ja5c02846_si_001.pdf [file ja5c02846_si_001.pdf]

Supplementary Information for Manuscript titled **Computer-Generated, Mechanistic Networks Assist in Assigning the Outcomes of Complex Multicomponent Reactions**

*Maciej Krzeszewski[1], Olena Vakuliuk[1], Mariusz Tasior[1], Agnieszka Wołos[1,3], Rafał Roszak[1,3], Karol Molga[1,3], Mohammad B. Teimouri[1,2] *, Bartosz A. Grzybowski[1,4,5] * and Daniel T. Gryko[1] **

AUTHOR ADDRESS

[1] Institute of Organic Chemistry, Polish Academy of Sciences, ul. Kasprzaka 44/52, Warsaw 01-224, Poland

[2] Faculty of Chemistry, Kharazmi University, South Mofateh Ave., Tehran 15719-14911, Iran

[3] Allchemy, Inc., 45th Street #201, Highland, IN 46322, USA

[4] IBS Center for Algorithmic and Robotized Synthesis (CARS), 50, UNIST-gil, Eonyang-eup, Ulju-gun, Ulsan 689-798, South Korea

[5] Department of Chemistry, UNIST, 50, UNIST-gil, Eonyang-eup, Ulju-gun, Ulsan 689-798, South Korea

*Corresponding authors. E-mail: daniel.gryko@icho.edu.pl (D.T.G.), nanogrzybowski@gmail.com (B. A. G.), teimouri@khu.ac.ir (M.B.T.)

This PDF file includes: Figures S1 to S28 Schemes S1 to S5, Tables S1 to S2 as well as references S1 to S7.

Section 1. Setting up calculations described in this publication.

All calculations were performed using MECH module of Allchemy platform. Calculations were set up in the "*New search*" tab, where settings (i) – (iv) (shown in **Figure S1**) were defined before launching the calculations:

(i) Introduction of substrates using "*from text*" option (as list of SMILES separated by dots).

(ii) Defining number of synthetic generations – this number translates into the number of mechanistic steps along the longest linear pathway.

(iii) Specifying reaction conditions (acidic-basic, temperature range, classes of solvents). In most calculations (see details below), default settings were applied.

(iv) Setting up limit for molecular weight of the generated molecules – custom limits for calculations were defined for each search based on the MS-observed masses.

(v) After settings (i) – (iv) were defined, calculations were started upon clicking on the "*Search*" button. Calculations took between 10 and 20 minutes.

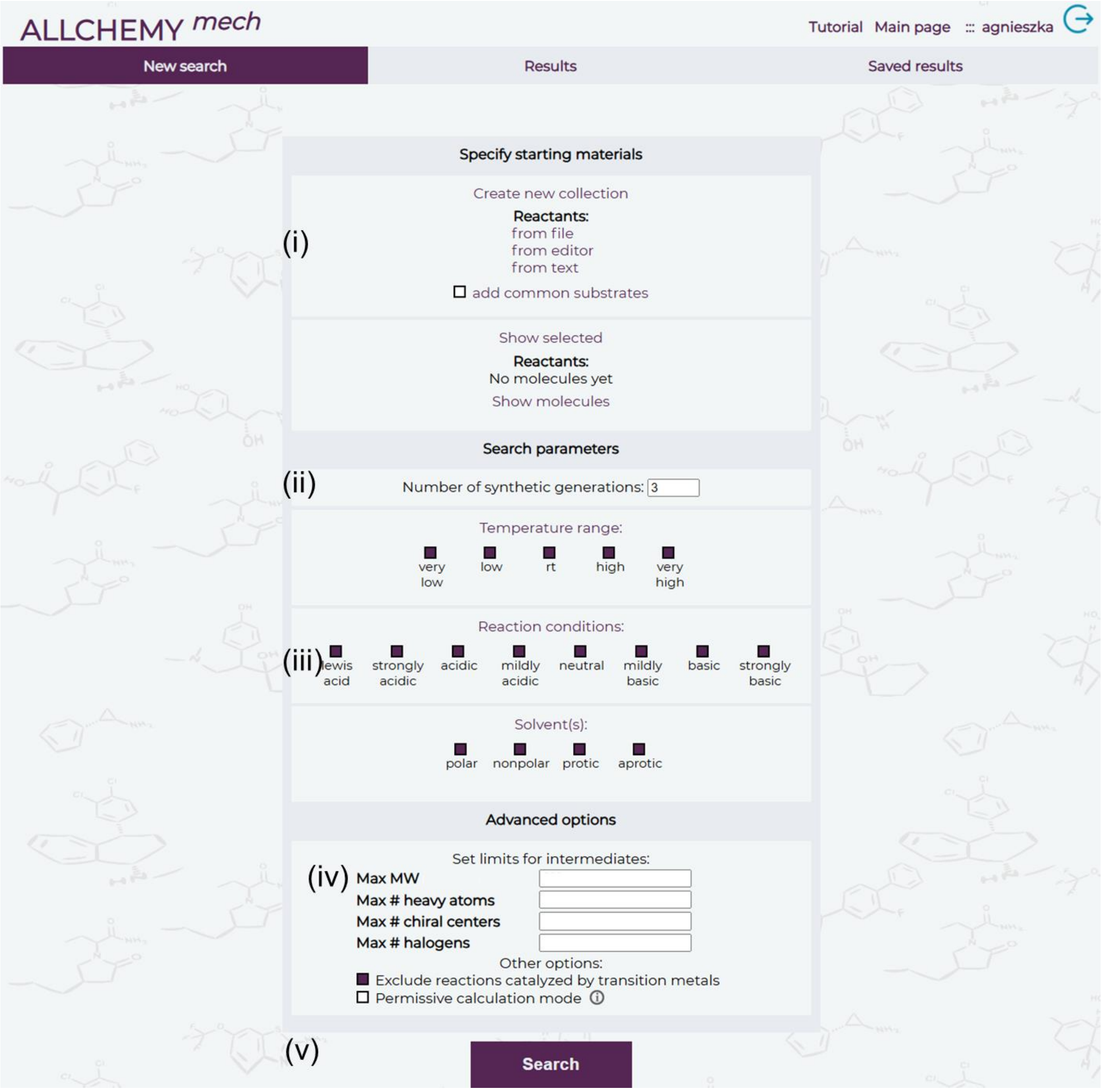

Figure S1. New search tab of Allchemy's MECH module. Setting up calculations described in this publication required: (i) introduction of starting materials, here "from text" as a list of SMILES separated by dots; (ii) setting up the number of synthetic generations, which translates into the number of mechanistic steps along the longest linear pathway; (iii) Setting up limits for calculations – possibly narrowing down admissible reaction conditions; (iv) excluding compounds exceeding molecular weight limit; and (v) Commencing the calculations by clicking on the "*Search*" button.

Section S.1.1. Search settings of calculations described in this publication.

Calculations for 4a

(*i*) Starting materials as list of SMILES separated by dots: CC(=O)C(C)=O.CC(C)(C)c1ccc(N)c(Br)c1.Cc1ccc2c(c1)cc(C=O)c1nnnn12

(*ii*) Number of generations: 7

(*iii*) default settings

(*iv*) Max MW: 1300

Calculations for <u>4b</u>

(*i*) Starting materials as list of SMILES separated by dots: CC(=O)C(C)=O.N#Cc1ccc(C=O)cc1.Nc1cccc2cc3ccccc3cc12

(*ii*) Number of generations: 9

(*iii*) default settings

(*iv*) Max MW: 700

Calculations for <u>4c</u>

(*i*) Starting materials as list of SMILES separated by dots: CC(=O)C(C)=O.Nc1cccc2ccccc12.CC(C)(C)c1cc(Br)c(C=O)c(-c2ccccc2)c1

(*ii*) Number of generations: 7

(*iii*) default settings

(*iv*) Max MW: 1200

Calculations for <u>4d</u>

(*i*) Starting materials as list of SMILES separated by dots: CC(=O)C(C)=O.Cn1c(C=O)cc2ccccc21.CC(C)(C)c1cc(N)cc(C(C)(C)C)c1

(*ii*) Number of generations: 6

(*iii*) default settings

(*iv*) Max MW: 900

Calculations for Figure 4

a) Starting from all three substrates

(*i*) Starting materials as list of SMILES separated by dots: CC(=O)C(C)=O.Cn1c(C=O)cc2ccccc21.CC(C)(C)c1cc(N)cc(C(C)(C)C)c1

(*ii*) Number of generations: 6

(*iii*) Temperature: high; Reaction conditions: strongly acidic, acidic, mildly acidic

(*iv*) Max MW: 900

b) Starting only from two substrates required for the synthesis of 4d

(*i*) Starting materials as list of SMILES separated by dots: Cn1c(C=O)cc2ccccc21.CC(C)(C)c1cc(N)cc(C(C)(C)C)c1

(*ii*) Number of generations: 6

(*iii*) Temperature: high; Reaction conditions: strongly acidic, acidic, mildly acidic

(*iv*) Max MW: 900

Section S2. Additional Allchemy screenshots of mechanistic pathways.

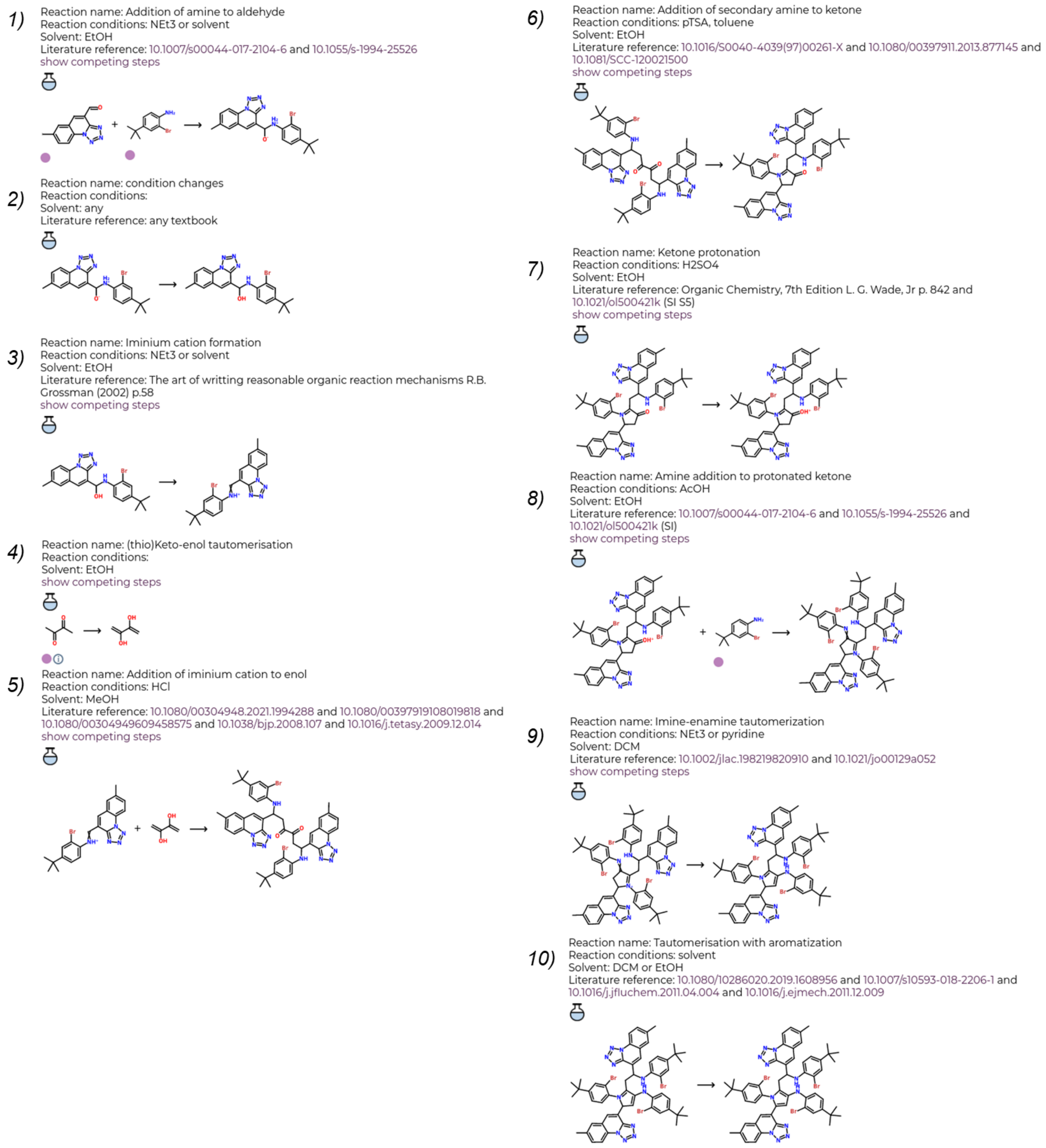

Figure S2. A longer mechanistic pathway leading to compound <u>4a</u> from Figure 2). This list of mechanistic steps is a screenshot from Allchemy's MECH module. Every step is accompanied by name, typical reaction conditions, solvent, and hyperlinks to illustrative literature references. By-products of each reaction can be visualized by clicking on the "flask" icon. Here, formation fo the first iminium cation (steps 1-3) is written out step-by-step, whereas the shortest pathway in the main text contains a two-step "shortcut" (in MECH, such shortcuts

are coded to accelerate network expansion, which is especially useful for very large networks, see ref. [21] for details).

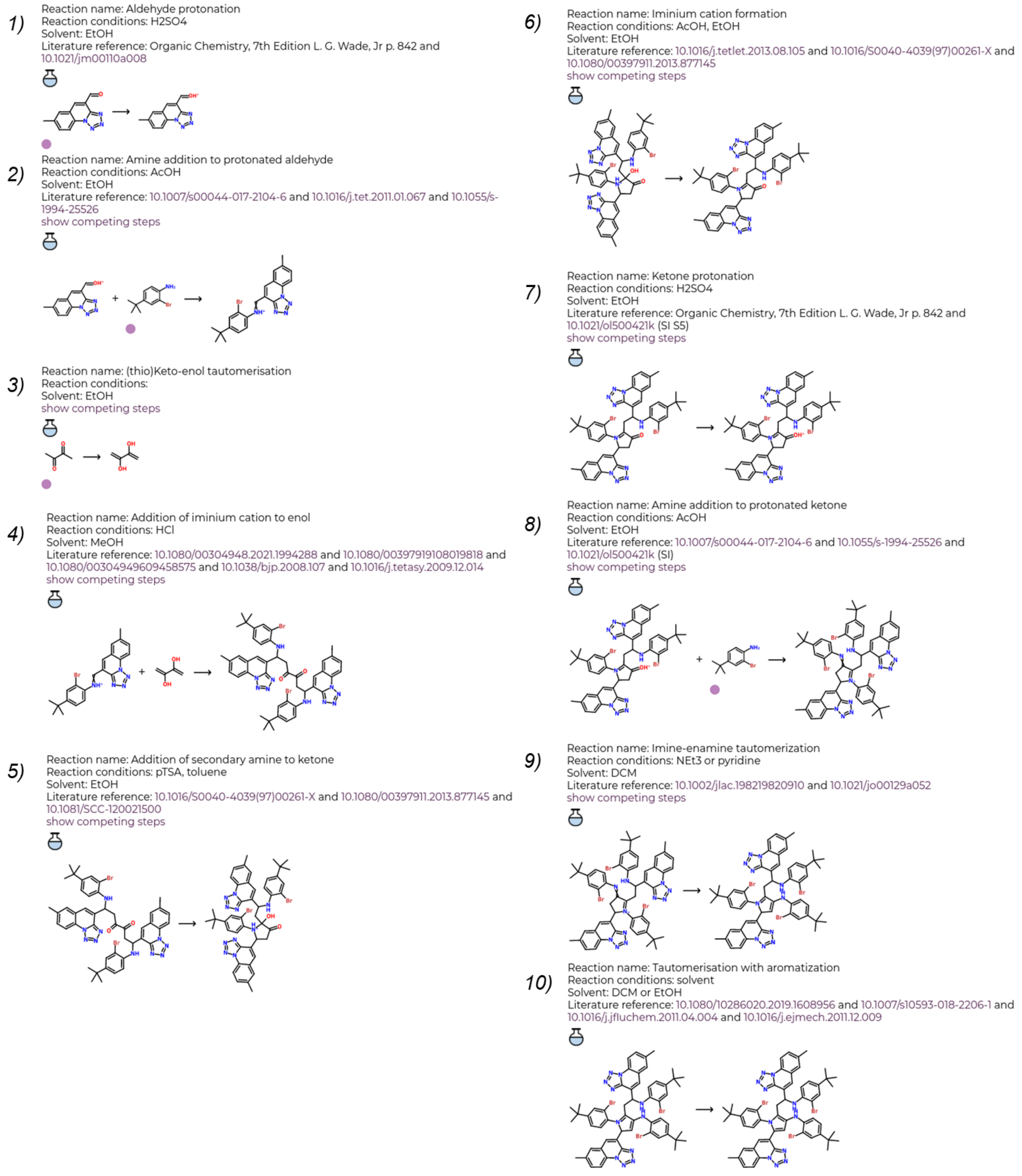

Figure S3. Another longer mechanistic pathway leading to compound 4a from Figure 2. This list of mechanistic steps is a screenshot from Allchemy's MECH module. Formation of the second iminium cation (steps 5-6) is depicted step-by-step, while the shortest pathway contains a one-step shortcut.

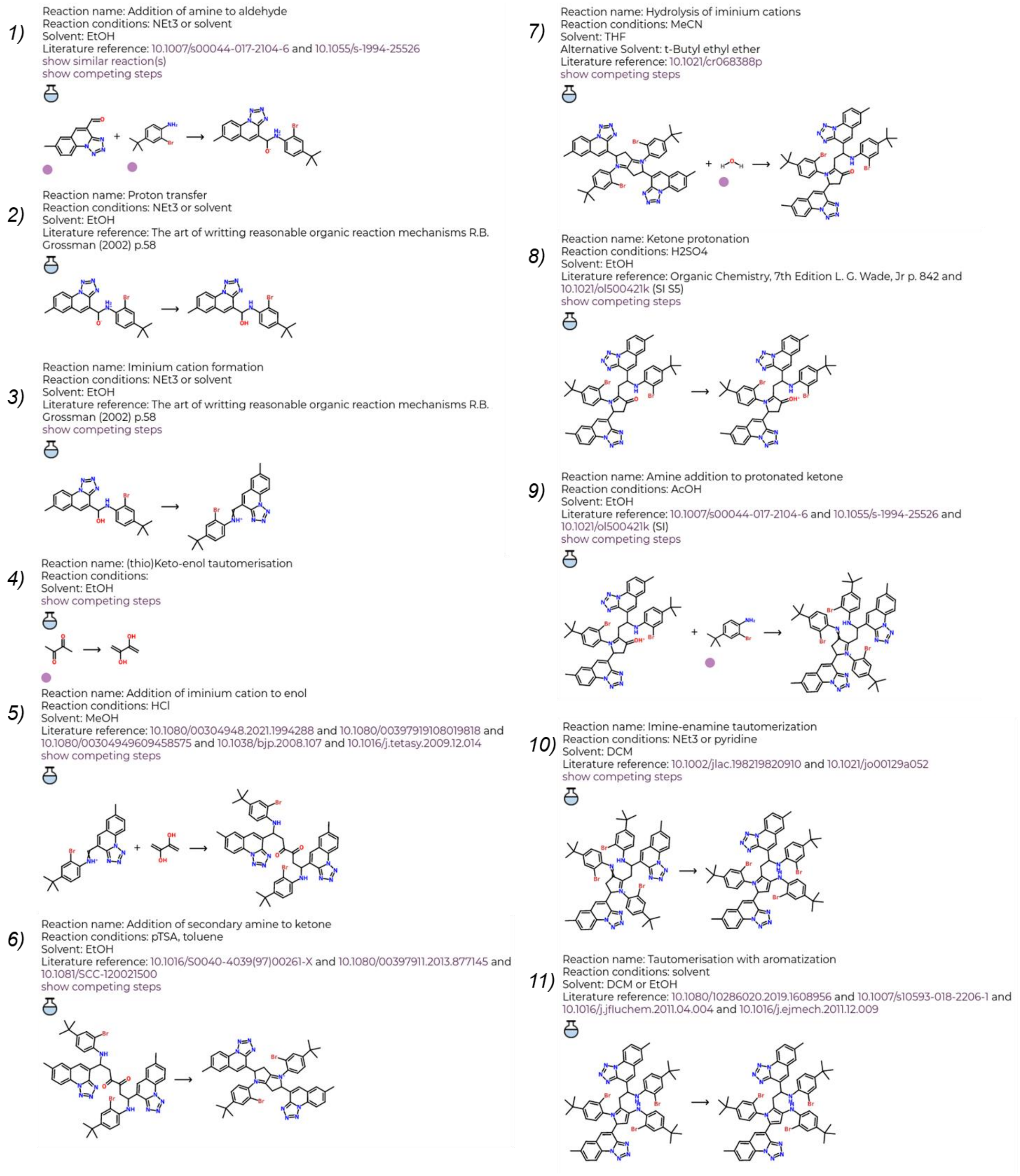

Figure S4. Another longer, mechanistic pathway leading to compound 4a from Figure 2. This list of mechanistic steps is a screenshot from Allchemy's MECH module. Formation of the first iminium cation (steps 1-3) is depicted step-by-step, while the shortest pathway contains a two-step shortcut. Furthermore, in step 6, both ketones form iminium cations, but in step 7, one of them undergoes hydrolysis.

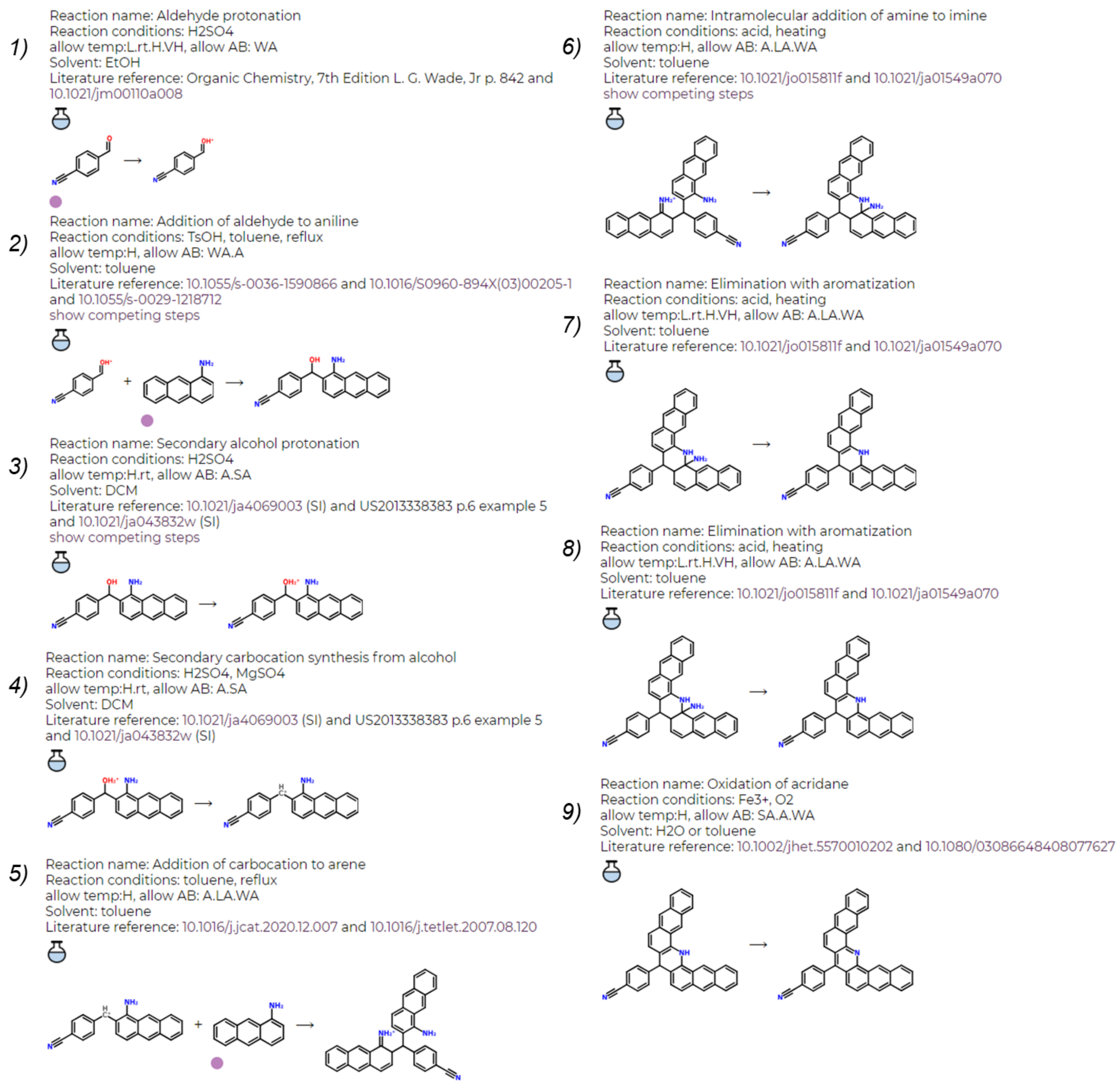

Figure S5. Mechanistic pathway leading to compound <u>4b</u> from Figure 3a. This list of mechanistic steps is a screenshot from Allchemy's MECH module.

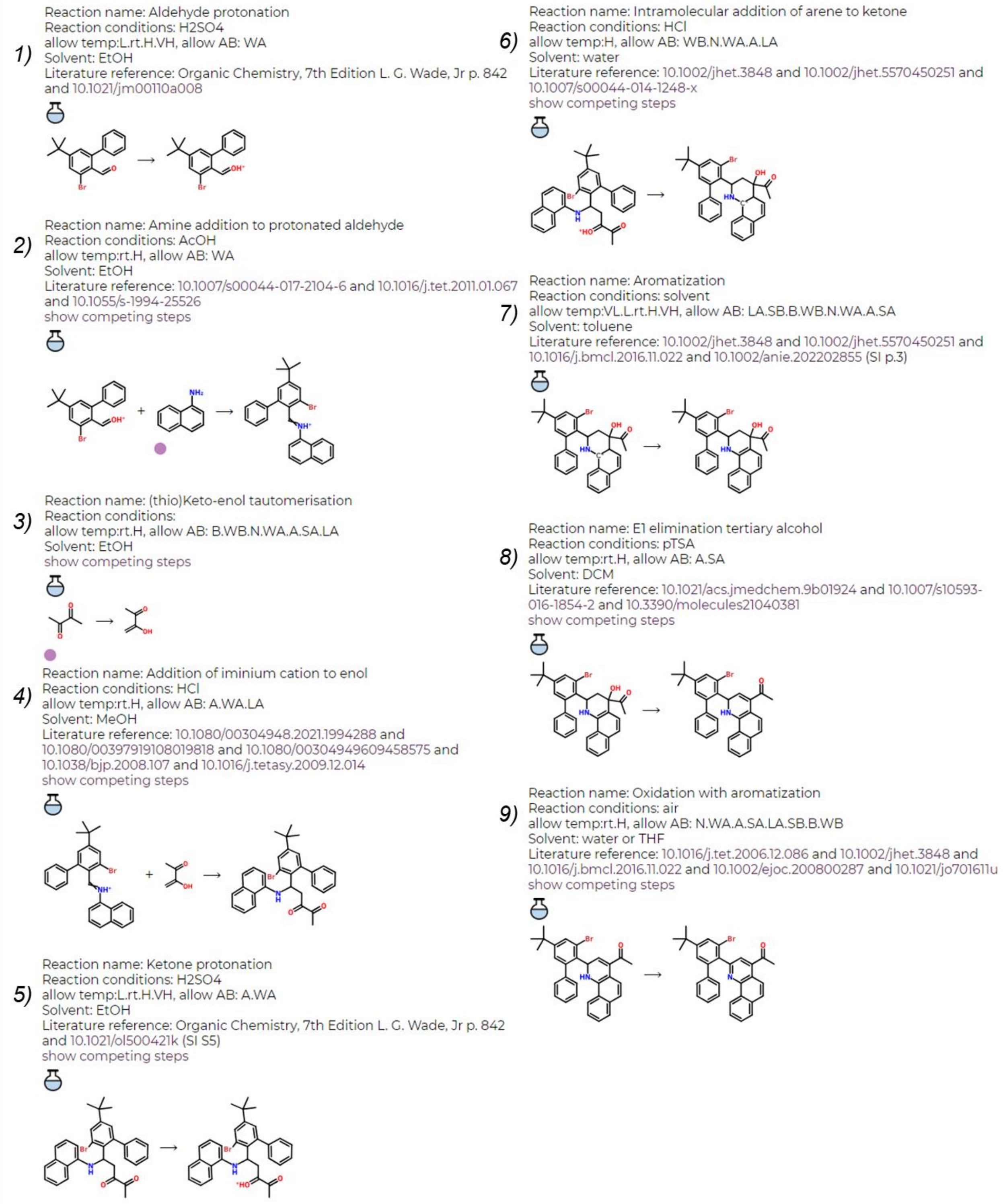

Figure S6. Mechanistic pathway leading to compound 4c from Figure 3b. This list of mechanistic steps is a screenshot from Allchemy's MECH module.

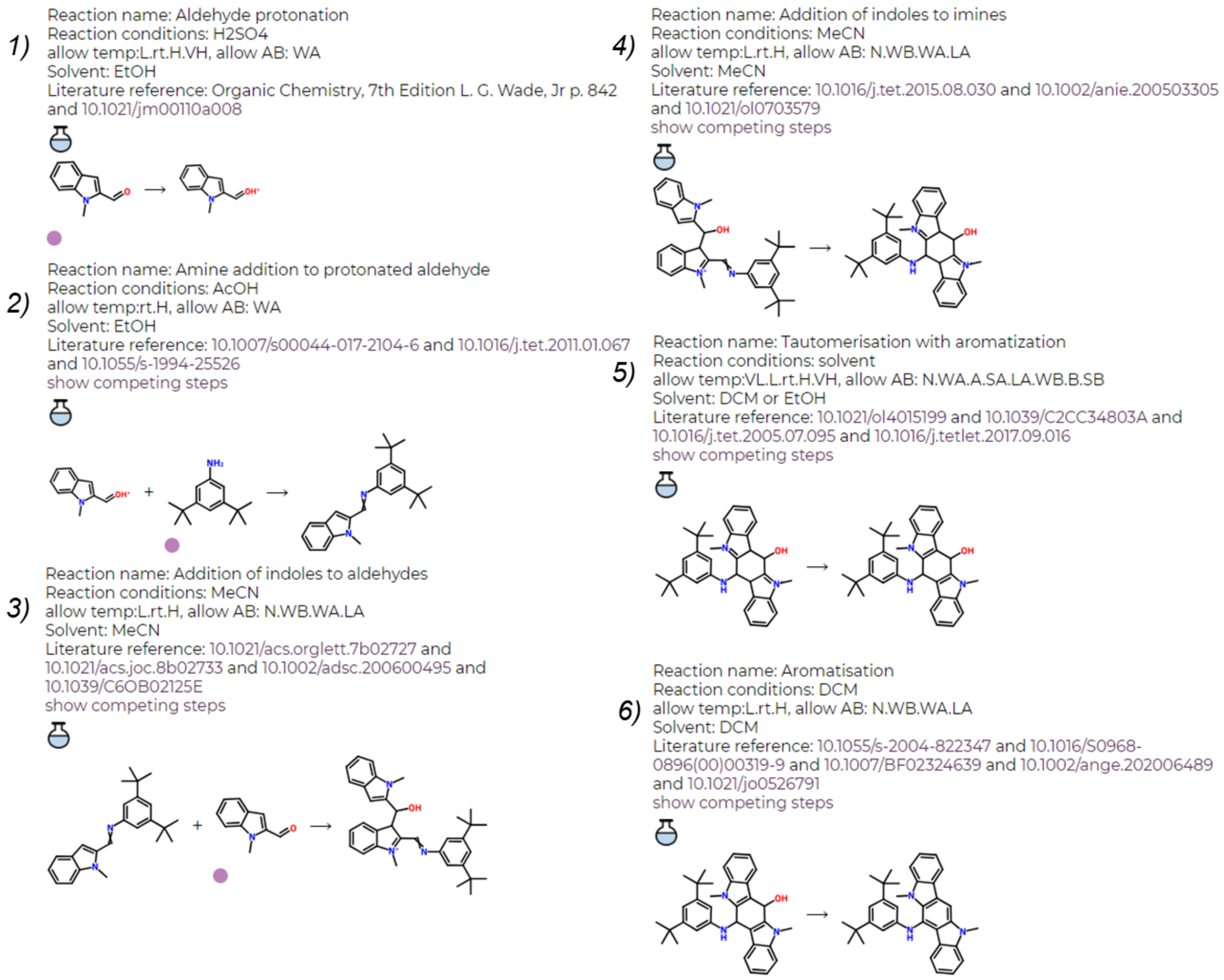

Figure S7. Mechanistic pathway leading to compound <u>4d</u> from Figure 3c. This list of mechanistic steps is a screenshot from Allchemy's MECH module.

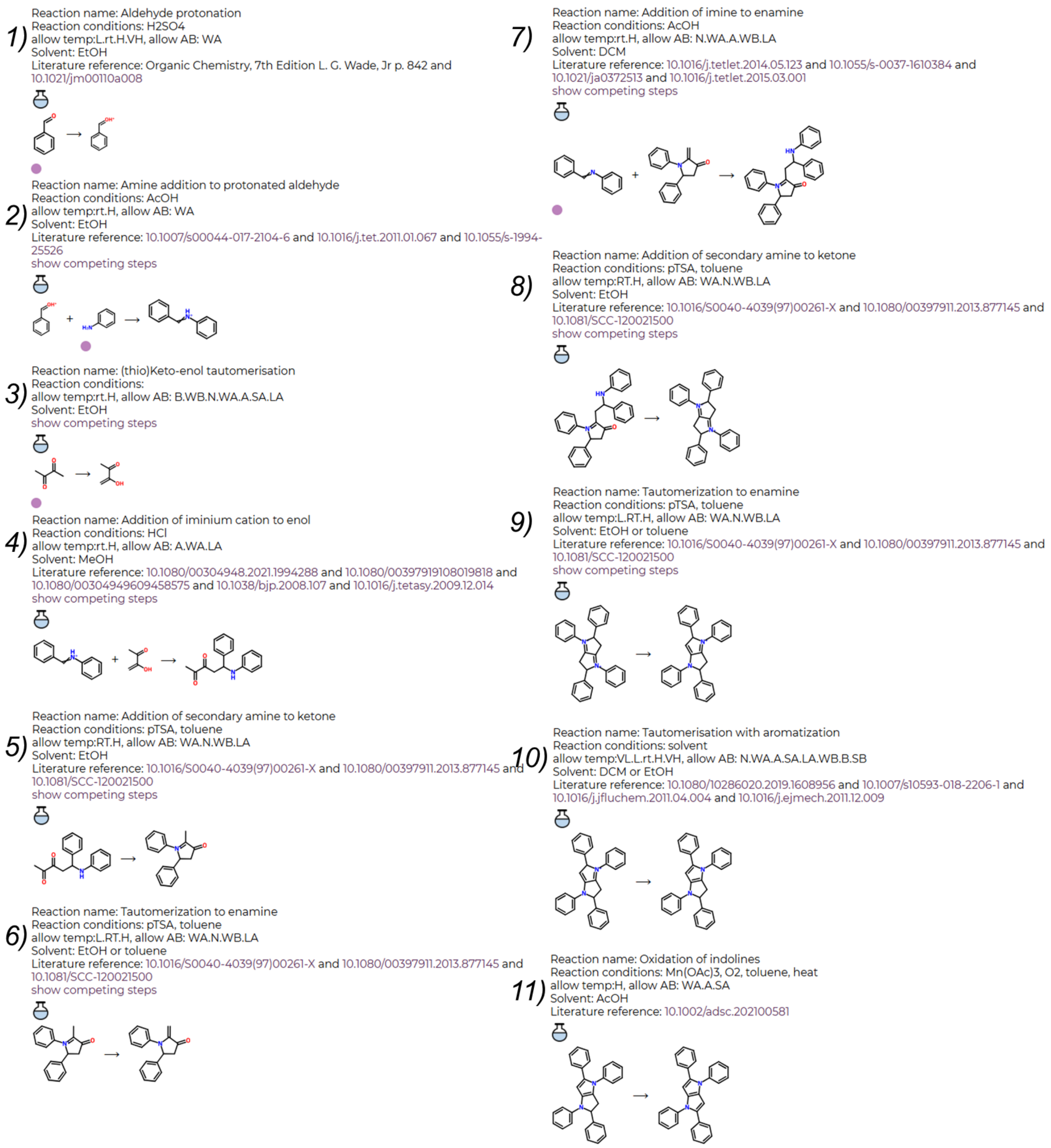

Figure S8. Allchemy screenshot of the mechanistic route leading to the DHPP scaffold, starting from benzaldehyde, aniline and diacetyl.

Section S3. Experimental and Metchods: Synthesis and characterization details

Section S3.1. General remarks.

All reagents and solvents were purchased from commercial sources and were used as received unless otherwise noted. Reagent-grade solvents (CH_2Cl_2, hexanes) were distilled before use. For water-sensitive reactions, solvents were dried using the Solvent Purification System from MBraun (https://www.mbraun.com/us/). Transformations with moisture and oxygen-sensitive compounds were performed under a stream of argon. The reaction progress was monitored using thin layer chromatography (TLC), which was performed on aluminum foil plates, covered with Silica gel 60 F254 (Merck) or Aluminium oxide 60 F254 (neutral, Merck). Product purification was done by means of column chromatography with Kieselgel 60 (Merck) or Aluminium oxide (Fluka). Occasionally, dry column vacuum chromatography (DCVC) for purification of products obtained was performed using Silica gel Type D 5F. The identity and purity of prepared compounds were confirmed by ^{1}H NMR and ^{13}C NMR spectrometry as well as by MS-spectrometry (via EI-MS or ESI-MS). NMR spectra were measured on Bruker AM 500 MHz, Bruker AM 600 MHz, Varian 600 MHz, instruments with TMS as the internal standard. Chemical shifts for ^{1}H NMR are expressed in parts per million (ppm) relative to tetramethylsilane (δ 0.00 ppm), $CDCl_3$ (δ 7.26 ppm), or DMSO (δ 2.50 ppm), pyridine-d_5 (δ 8.74, 7.58, 7.22 ppm), THF-d_4 (δ 3.58, 1.73 ppm) or TCE-d_2 (δ 6.00 ppm). Chemical shifts for ^{13}C NMR are expressed in ppm relative to $CDCl_3$ (δ 77.0 ppm), DMSO (δ 39.5 ppm), pyridine-d_5 (δ 150.4, 135.9, 123.9 ppm), THF-d_4 (δ 67.5, 25.3 ppm) or TCE-d_2 (δ 73.8 ppm). Data are reported as follows: chemical shift, multiplicity (s = singlet, d = doublet, dd = doublet of doublets, ddd = doublet of doublet of doublets, t = triplet, td = triplet of doublets, q = quartet, m = multiplet), coupling constant (Hz), and integration. All melting points for crystalline products were measured with the automated melting point apparatus EZ-MELT and were given without correction.

Compounds 2,6-dibromo-4-*tert*-butylaniline (**2.2.2**)[S1] and 1,3-dibromo-5-*tert*-butyl-benzene (**S2.2.3**)[S1] were prepared according to the literature procedures.

Section S3.2. Synthetic procedures.

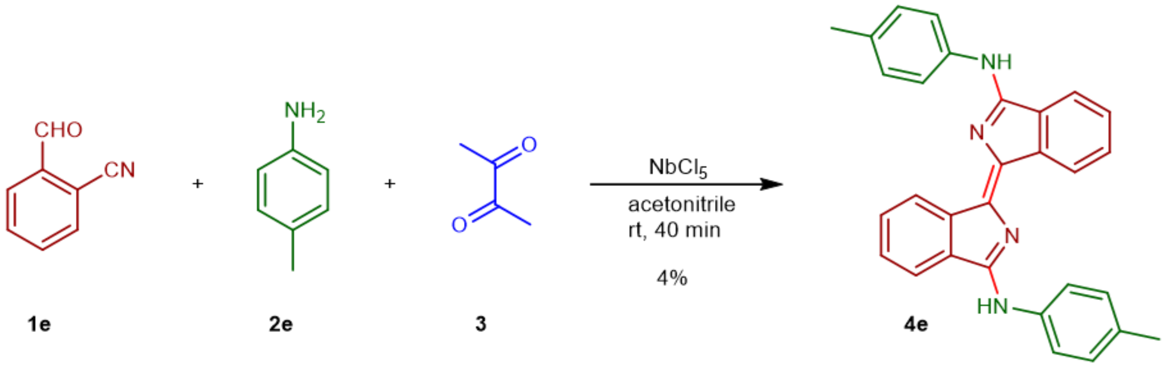

Scheme S1. Reaction scheme of the reaction leading to an unexpected product of a known MCR (compound **4e**) described in main-text **Figure 3d**.

(E)-*N*^{3},*N*$^{3'}$-di-*p*-tolyl-[1,1'-biisoindolylidene]-3,3'-diamine (4e)

$NbCl_5$ (68 mg, 0.25 mmol) was dissolved in dry acetonitrile (1 mL) under argon atmosphere. 2-Cyanobenzaldehyde (**1e**, 262 mg, 2 mmol) and *p*-toluidine (**2e**, 214 mg, 2 mmol) in 5 mL of dry acetonitrile were added and the resulting mixture was stirred at room temperature for 40 minutes. Reaction mixture was quenched with water (3 mL), extracted with DCM (3 × 10 mL), combined organic phases were washed with sat. $NaHCO_3$, water and brine and dried with $MgSO_4$. The solvent was removed and the residue was purified by column chromatography (alumina, DCM). Crystallization from DCM/hexanes afforded pure **4e** (35 mg, 4%) as an orange-red solid. ***Reaction performed with the addition of diacetyl 3 (3, 88 µL, 1 mmol) leads to the same outcome.*** M.p. 300 °C (decomp.). ^{1}H NMR (500 MHz, DMSO-d_6) δ 9.99 (s, 2H, NH), 8.80 (d, *J* = 7.5 Hz, 2H, ABCD), 8.22 (d, *J* = 7.5 Hz, 2H, ABCD), 8.13 (d, *J* = 8.5 Hz, 4H, AA'XX'), 7.63 (dt, *J* = 8.0, 1 Hz, 2H, ABCD), 7.52 (dt, *J* = 7.5, 1.5 Hz, 2H, ABCD), 7.31 (d, *J* = 8.0 Hz, 4H, AA'XX'), 2.37 (s, 6H, CH_3) ppm. ^{13}C{^{1}H} NMR (126 MHz, DMSO-d_6) δ 159.4, 142.5, 139.7, 138.6, 133.8, 131.8, 129.8, 129.3, 127.5, 125.3, 120.8, 119.6, 21.0. HRMS (ESI): m/z calcd for $C_{30}H_{25}N_4$: 441.2079 $[M+H]^+$; found: 441.2064.

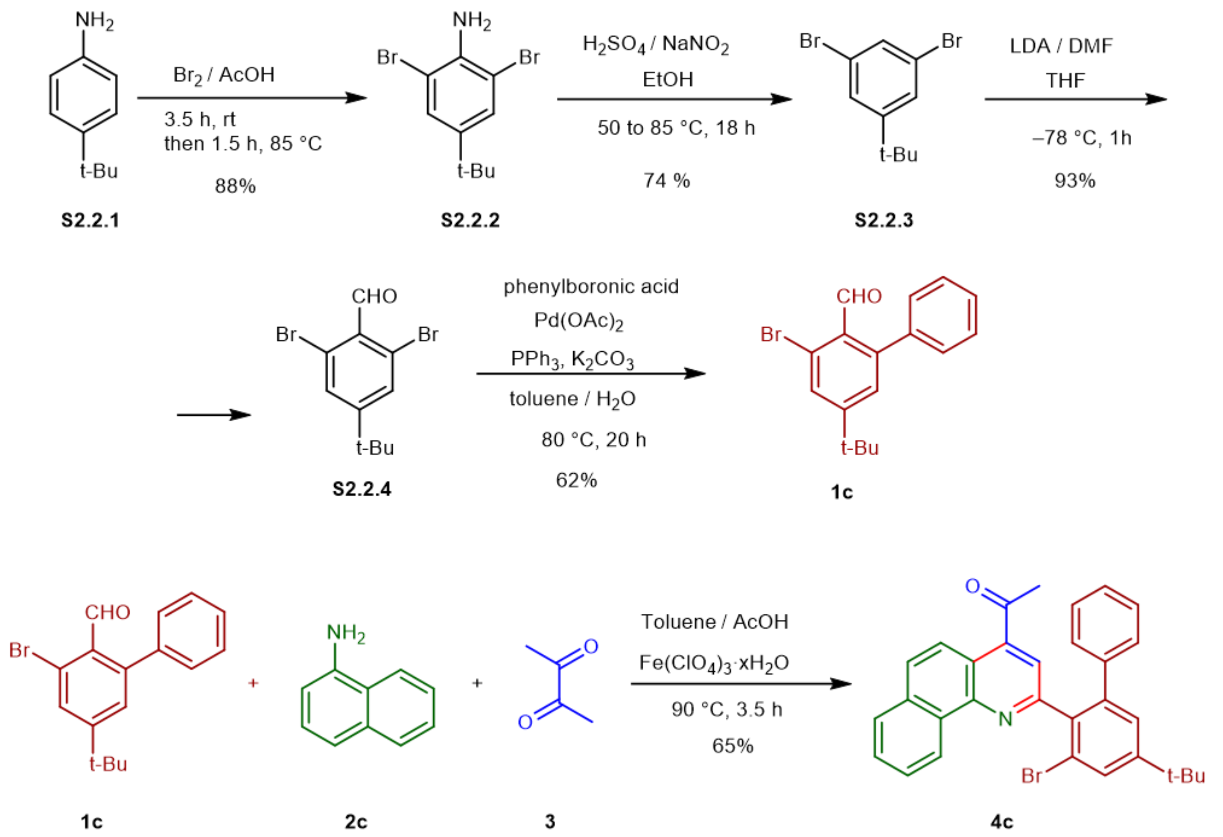

Scheme S2. Reaction scheme of the synthesis of aldehyde substrate **1c** (upper) and an unexpected product of a known MCR (compound **4c**, lower) described in main-text **Figure 3b**.

2,6-Dibromo-4-(*tert*-butyl)benzaldehyde (S2.2.4). LDA (20.5 mL, 41 mmol) was added dropwise at -78 °C to the solution of 1,3-dibromo-5-*tert*-butyl-benzene (**S2.2.3**, 9.9 g, 34 mmol) in dry THF (100 mL). The mixture was stirred for 30 min at -78 °C, and then DMF (3.2 mL, 41 mmol) was added dropwise while maintaining the temperature at - 78 °C. A purple solution was stirred for an additional 30 min at -78 °C. Then the solution was left to warm to room temperature and hydrolyzed with 10% H_2SO_{4aq} (100 mL). The yellow organic layer was separated. The water layer was extracted with diethyl ether (3 × 100 mL), and the extract was added to the organic phase. Solvents were evaporated to leave the crude product which was purified by column chromatography on silica gel (hexanes/DCM, 2:1) affording **S2.2.4** (9.2 g, 93%) as a yellowish solid. M.p. 67−68 °C. ^{1}H NMR (500 MHz, $CDCl_3$) δ 10.25 (s, 1H, CHO), 7.63 (s, 2H, Ar), 1.33 (s, 9H, *t*-Bu) ppm. ^{13}C{^{1}H} NMR (126 MHz, $CDCl_3$) δ 191.1, 158.8, 131.3, 129.9, 125.2, 35.5, 30.9 ppm. HRMS (EI): m/z calcd for $C_{11}H_{12}OBr_2^{\cdot+}$: 317.9249 [M]$^{\cdot+}$; found: 317.9263.

3-Bromo-5-(*tert*-butyl)-[1,1'-biphenyl]-2-carbaldehyde (1c). 2,6-Dibromo-4-(*tert*-butyl)benzaldehyde (**S2.2.4**, 4.8 g, 15 mmol), phenylboronic acid (2.0 g, 16.5 mmol), K_2CO_3 (4.1 g, 30 mmol), PPh_3 (0.8 g, 3 mmol) and $Pd(OAc)_2$ (0.34 g, 1.5 mmol) were placed in a 100 mL Schlenk flask, which was flushed with argon before use. Then 45 mL of 1:1 v/v mixture of toluene and water was added, and the resulting mixture was stirred at 80 °C for 16 h. After cooling two layers were separated, aqueous layer was extracted with ethyl acetate (3 × 20 mL). Organic layers were combined, solvents were evaporated and the crude product was purified by column chromatography (SiO_2, hexanes/ethyl acetate, 9:1 to 6:1) affording **1c** (3.0 g, 62%) as an off-white solid. M.p. 66−68 °C. ^{1}H NMR (500 MHz, $CDCl_3$) δ 9.99 (s, 1H, CHO), 7.70 (d, *J* = 1.7 Hz, 1H, Ar), 7.45 – 7.42 (m, 3H, Ph), 7.33 (d, *J* = 1.7 Hz, 1H, Ar), 7.31 – 7.28 (m, 2H, Ph), 1.36 (s, 9H, *t*-Bu) ppm. ^{13}C{^{1}H} NMR (126 MHz, $CDCl_3$) δ 191.9, 157.2, 146.8, 138.9, 131.1, 130.5, 129.7, 128.5, 128.2, 127.8, 123.5, 35.4, 31.1 ppm. HRMS (ESI): m/z calcd for $C_{17}H_{17}OBrNa^+$: 339.0355 [M+Na]$^+$; found: 339.0366.

1-(2-(3-bromo-5-(*tert*-butyl)-[1,1'-biphenyl]-2-yl)benzo[h]quinolin-4-yl)ethan-1-one (4c). Glacial acetic acid (1 mL), toluene (1 mL), 2,6-dibromo-4-(*tert*-butyl)benzaldehyde (**1c**, 317 mg, 1 mmol), and 1-naphthylamine (**2c**, 143 mg, 1 mmol) were placed in a 25 mL round-bottom flask equipped with a magnetic stir bar. The mixture was stirred at 90 °C for 30 min. After that time, $Fe(ClO_4)_3{\cdot}xH_2O$ (11 mg, 0.03 mmol) was added, followed by diacetyl (**3**, 44 µL, 0.5 mmol). The resulting mixture was stirred at 90 °C (oil bath) in an open flask under air for 3 hours. Solvents were evaporated and the crude product was purified by column chromatography (SiO_2, hexanes/DCM, 3:2 to 1:1) affording **4c** (166 mg, 65%) as a yellow solid. M.p. 178−180 °C. ^{1}H NMR (600 MHz, $CDCl_3$) δ 9.29 – 9.25 (m, 1H, Benzoquinoline), 8.31 (d, *J* = 9.2 Hz, 1H, Benzoquinoline), 7.92 – 7.89 (m, 1H, Benzoquinoline), 7.88 (d, *J* = 9.2 Hz, 1H, Benzoquinoline), 7.82 (d, *J* = 1.9 Hz, 1H, Ar), 7.74 – 7.68 (m, 2H, Benzoquinoline), 7.50 (d, *J* = 1.9 Hz, 1H, Ar), 7.41 (s, 1H, Benzoquinoline), 7.15 – 7.12 (m, 2H, Ph), 7.11 – 7.07 (m, 3H, Ph), 2.44 (s, 3H, CH_3), 1.44 (s, 9H, *t*-Bu) ppm. ^{13}C{^{1}H} NMR (126 MHz, $CDCl_3$) δ 201.6, 156.8, 153.6, 147.1, 143.6, 141.6, 141.3, 137.1, 133.5, 131.6, 129.9, 129.8, 129.7, 128.7, 128.1, 127.6, 127.3, 127.1, 126.9, 125.5, 123.8, 123.5, 122.3, 120.4, 35.1, 31.4, 30.0 ppm. HRMS (ESI): m/z calcd for $C_{31}H_{27}NOBr^+$: 508.1271 [M+H]$^+$; found: 508.1279.

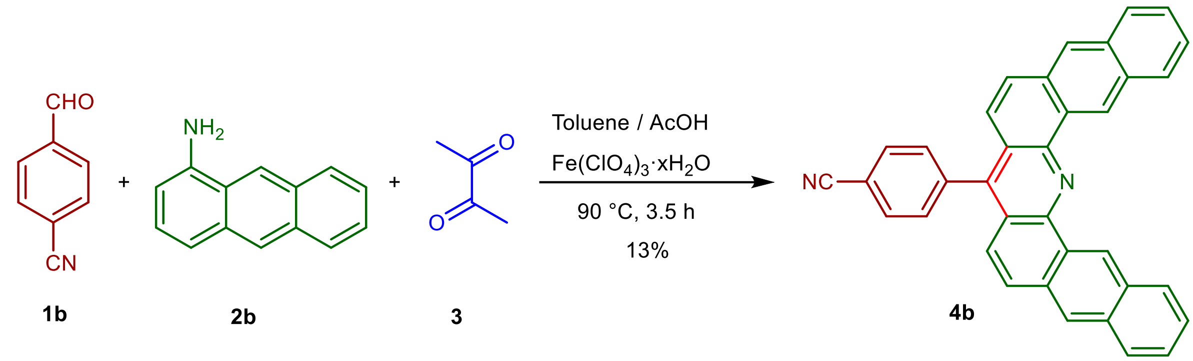

1b 2b 3 4b

Scheme S3. Reaction scheme of the reaction leading to unexpected product of a known MCR (compound **4b**) described in main-text **Figure 3a**.

4-(dinaphtho[2,3-c:2',3'-h]acridin-8-yl)benzonitrile (**4b**). Glacial acetic acid (1.5 mL), toluene (1.5 mL), 4-cyanobenzaldehyde (**1b**, 262 mg, 2 mmol), and 1-anthrylamine (**2b**, 193 mg, 1 mmol) were placed in a 25 mL round-bottom flask equipped with a magnetic stir bar. The mixture was stirred at 90 °C for 30 min. After that time, $Fe(ClO_4)_3$·x H_2O (21 mg, 0.06 mmol) was added. The resulting mixture was stirred at 90 °C (oil bath) in an open flask under air for an additional 3 hours. Solvents were evaporated and the crude product was filtered through the pad of SiO_2 (hexanes/DCM, 1:1) affording **4b** (95.9 mg, 20%) as a yellow solid (recrystallized from DCM/hexanes. ***Reaction performed with the addition of diacetyl 3 (3, 88 µL, 1 mmol) and with reagents ratio aldehyde: aniline = 1:1 at 90 °C for 3 h leads to the same product at 13% yield***. M.p. 372−374 °C. ^{1}H NMR (600 MHz, $CDCl_3$) δ 10.33 (s, 2H), 8.47 (d, *J* = 8.1 Hz, 2H, Ar), 8.39 (s, 2H, Ar), 8.12 (d, *J* = 8.0 Hz, 2H, Ar), 7.98 – 7.93 (m, 2H, AA'XX'), 7.76 (d, *J* = 9.3 Hz, 2H, Ar), 7.71-7.69 (m, 2H, Ar), 7.68-7.65 (m, 2H, Ar), 7.64 – 7.62 (m, 2H, AA'XX'), 7.24 (d, *J* = 9.2 Hz, 2H, Ar). ^{13}C{^{1}H} NMR (151 MHz, $CDCl_3$) δ 146.2, 142.7, 141.8, 133.3, 132.4, 131.4, 131.3, 130.0, 129.4, 128.7, 128.0, 126.7, 126.6, 126.1, 125.2, 123.3, 122.7, 118.6, 112.4 ppm. HRMS (ESI): m/z calcd for $C_{36}H_{21}N_2^+$: 481.1699 $[M+H]^+$; found: 481.1711.

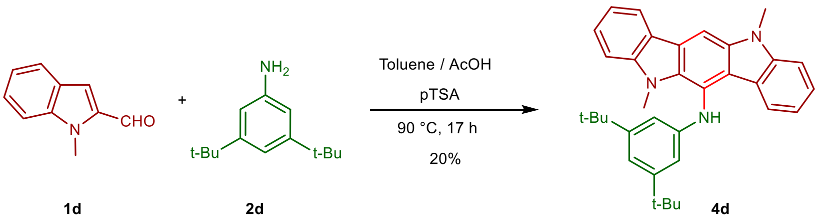

1d 2d 4d

Scheme S4. Reaction scheme of the reaction leading to an unexpected product of a known MCR (compound **4d**) described in main-text **Figure 3c**.

N-(3,5-di-*tert*-butylphenyl)-5,11-dimethyl-5,11-dihydroindolo[3,2-b]carbazol-6-amine (4d). Glacial acetic acid (2 mL), toluene (2 mL), 1-methyl-1*H*-indole-2-carbaldehyde (**1d**, 636 mg, 4 mmol), 3,5-di-*tert*-butylaniline (**2d**, 410 mg, 2 mmol) and *p*TSA (69 mg, 20 mol%) were placed in a 25 mL round-bottom flask equipped with a magnetic stir bar. The mixture was stirred at 90 °C for 17 h in an open flask under air. The precipitate was filtered off, washed with cold acetonitrile and recrystallized from hot THF, affording **4d** (195 mg, 20%) as a yellow solid. ***Reaction performed with the addition of diacetyl 3 (3, 88 µL, 1 mmol) and with reagents ratio aldehyde: aniline = 1:1 at 90 °C for 3 h leads to the same product at 2% yield.*** M.p. >290 °C (decomp.).^{1}H NMR (600 MHz, THF-d_4) δ 8.18 (d, J = 7.7 Hz, 1H), 8.10 (d, J = 7.7 Hz, 1H), 8.05 (bs, 1H), 7.40-7.36 (m, 3H), 7.32 (dt, J_1 = 8.1 Hz, J_2 = 1.0 Hz, 1H), 7.27 (bs, 1H), 7.16-7.15 (m, 1H), 6.98-, 6.94 (m, 1H), 6.80-6.78 (m, 1H), 6.63 (bs, 2H), 4.00 (s, 3H), 3.95 (s, 3H), 1.15 (s, 18H); ^{13}C{^{1}H} NMR (151 MHz, THF-d_4) δ 152.3, 148.6, 144.3, 143.1, 138.6, 134.3, 126.4, 125.8, 125.6, 124.3, 124.1, 123.2, 121.8, 120.7, 120.6, 118.9, 118.5, 118.5, 109.1, 108.8, 108.2, 97.1, 35.3, 31.7, 31.6, 29.3; For the full signal assignment, see **Figure S11**; HRMS (EI): m/z calcd. for $C_{34}H_{37}N_3$: 487.2987 $[M]^{\cdot+}$; found: 487.2992.

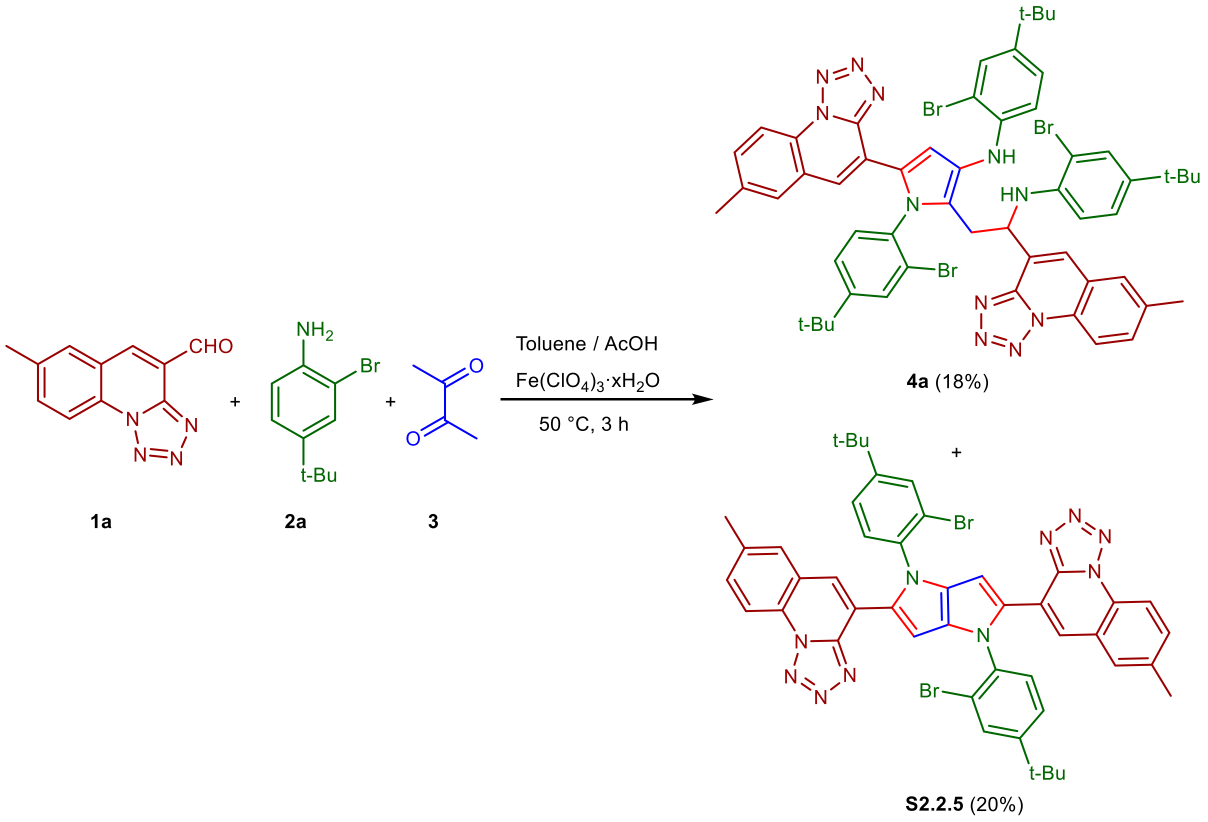

Scheme S5. Reaction scheme of the reaction leading to an unexpected product of a known MCR (compound **4a**) described in main-text **Figure 2**.

4,4'-(1,4-bis(2-bromo-4-(*tert*-butyl)phenyl)-1,4-dihydropyrrolo[3,2-*b*]pyrrole-2,5-diyl)bis(7-methyltetrazolo[1,5-*a*]quinoline) (4a) and **N,1-bis(2-bromo-4-(*tert*-butyl)phenyl)-2-(2-((2-bromo-4-(*tert*-butyl)phenyl)amino)-2-(7-methyltetrazolo[1,5-*a*]quinolin-4-yl)ethyl)-5-(7-methyltetrazolo[1,5-*a*]quinolin-4-yl)-1*H*-pyrrol-3-amine (S2.2.5)**. Glacial acetic acid (1 mL), toluene (1 mL), 7-methyltetrazolo[1,5-*a*]quinoline-4-carbaldehyde (**1a**, 212 mg, 1 mmol), and 2-bromo-4-(*tert*-butyl)aniline (**2a**, 228 mg, 1 mmol) were placed in a 25 mL round-bottom flask equipped with a magnetic stir bar. The mixture was stirred at 50 °C for 1 h. After that time, $Fe(ClO_4)_3{\cdot}xH_2O$ (11 mg, 0.03 mmol) was added, followed by diacetyl (**3**, 44 µL, 0.5 mmol). The resulting mixture was stirred at 50 °C (oil bath) in an open flask under air for 2 hours. After cooling to room temperature, the precipitate was filtered off, washed with cold methanol and diethyl ether and dried under vacuum affording **S2.2.5** (179 mg, 20%) as an orange solid. Next, all organic fractions were combined and concentrated in vacuo. Then, the resulting solid was recrystallized from acetone affording **4a** (203 mg, 18%) as a bright yellow solid.

N,1-bis(2-bromo-4-(*tert*-butyl)phenyl)-2-(2-((2-bromo-4-(*tert*-butyl)phenyl)amino)-2-(7-methyltetrazolo[1,5-*a*]quinolin-4-yl)ethyl)-5-(7-methyltetrazolo[1,5-*a*]quinolin-4-yl)-1*H*-pyrrol-3-amine (4a). Yellowish solid. Yield: 203 mg (18%). M.p. 192-193 °C. ^{1}H NMR (500 MHz, $CDCl_3$) δ 8.42 (d, *J* = 6.6 Hz, 1H, Ar), 8.40 (d, *J* = 6.6 Hz, 1H, Ar), 7.76 (d, *J* = 2.0 Hz, 1H, Ar), 7.68 (s, 1H, Ar), 7.64 (s, 1H, Ar), 7.56 (d, *J* = 8.4 Hz, 1H, Ar), 7.52 (s, 1H, Ar), 7.50 – 7.46 (m, 2H, Ar), 7.39 (d, *J* = 2.1 Hz, 1H, Ar), 7.20 (dd, *J* = 8.1, 1.7 Hz, 1H, Ar), 7.07 – 7.03 (m, 2H, Ar), 6.94 (dd, *J* = 8.5, 2.1 Hz, 1H, Ar), 6.82 (d, *J* = 7.9 Hz, 1H, Ar), 6.73 (d, *J* = 8.5 Hz, 1H, Ar), 6.63 (s, 1H, Pyrole), 6.25 (d, *J* = 8.6 Hz, 1H, Ar), 5.74 (s, 1H, NH), 5.69 (d, *J* = 7.6 Hz, 1H, CH-N*H*), 5.02 (m, 1H, CH_2-C*H*-NH), 3.44 (dd, *J* = 15.1, 7.3 Hz, 1H, CH_2-CH), 3.36 (dd, *J* = 15.2, 6.9 Hz, 1H, CH_2-CH), 2.52 (s, 3H, CH_3), 2.43 (s, 3H, CH_3), 1.43 (s, 9H, *t*-Bu), 1.27 (s, 9H, *t*-Bu), 1.18 (s, 9H, *t*-Bu); $^{13}C\{^1H\}$ NMR (126 MHz, $CDCl_3$) δ 155.1, 146.8, 146.1, 142.5, 141.8, 141.7, 140.8, 138.1, 138.0, 134.3, 132.1, 131.3, 131.2, 130.2, 129.8, 129.7, 129.4, 129.4, 128.7, 128.2, 127.8, 127.0, 126.7, 126.0, 125.9, 125.8, 125.3, 125.2, 125.0, 124.2, 123.9, 123.8, 117.5, 116.3, 116.1, 114.1, 113.3, 111.6, 110.3, 109.6, 55.2, 35.1, 34.0, 33.9, 31.4, 31.3, 31.2, 30.4, 21.4, 21.3 ppm. HRMS (APCI): m/z calcd for $C_{56}H_{57}Br_3N_{11}$: 1120.2349 $[M+H]^+$; found: 1120.2351.

4,4'-(1,4-bis(2-bromo-4-(*tert*-butyl)phenyl)-1,4-dihydropyrrolo[3,2-*b*]pyrrole-2,5-diyl)bis(7-methyltetrazolo[1,5-*a*]quinoline) (S2.2.5.). Orange solid (as a *mixture of atropoisomers*). Yield: 179 mg (20%). M.p. 300 °C (decomp.). ^{1}H NMR (600 MHz, TCE-d_2, 85 °C) δ 8.46 (2xd, *J* = 8.9 Hz, 2H, Ar), 7.83 (d, *J* = 2.1 Hz, 1H, Ar), 7.77 (d, *J* = 2.1 Hz, 1H, Ar), 7.61 (d, *J* = 8.2 Hz, 1H, Ar), 7.59-7.52 (m, 5H, Ar), 7.50-7.44 (m, 2H, Ar), 7.40 (d, *J* = 2.0 Hz, 1H, Ar), 7.38 (s, 1H, Ar), 7.21 (s, 1H, Ar), 7.19 (s, 1H, Ar), 6.92 (2xs, 2x1H, pyrrolopyrrole), 2.40 (s, 6H), 1.43, 1.42 (2xs, 2×9H, 2x*t*-Bu) ppm; Due to very low solublity, we were not able to measure ^{13}C NMR spectrum of this compound even at 85 °C in TCE-d_2. HRMS (ESI): m/z calcd for $C_{46}H_{42}Br_2N_{10}Na$: 915.1858 $[M+Na]^+$; found: 915.1823.

Section S3.3. Structure determination for compounds 4b, 4c, and 4d.

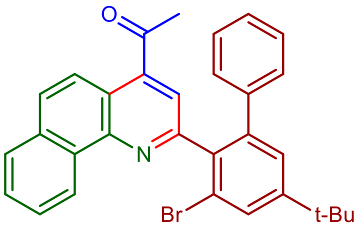

4c

High-resolution mass spectrometry determined the molecular formula of **4c** for 508.1279 peak as $C_{31}H_{27}NOBr^{+}$ $[M+H]^{+}$. The structure proposed by the algorithm (see main-text **Figure 3b**) is congruent with the experimentally recorded spectra. Specifically, the presence of a singlet at 2.44 ppm in the ^{1}H NMR spectrum and signal 201.6 ppm in the ^{13}C NMR spectra indicates one carbonyl/methyl group from parent butane-2,3-dione participated in the reaction. Moreover, the combination of 1D and 2D NMR spectra suggests the presence of moiety originating from the parent aldehyde. Therefore, the remaining part of the molecule is of the formula: $C_{13}H_{7}N$ ($C_{31}H_{26}NOBr$ - $C_{2}H_{3}O$ (*for acetyl*) – $C_{16}H_{16}Br$ (*for 3-bromo-5-(tert-butyl)-[1,1'-biphenyl]-2-yl*), which suggests a benzoquinoline structural motif.

The ^{1}H-^{1}H COSY correlations indicate that 7 remaining protons from the aromatic region of the spectra can be assigned to 2 spin systems [4H (ABCD) and 2H (AB)] along with the sole signal at 7.41 ppm. The latter one is the only signal having a strong correlation with the acetyl group and is thus assigned to a hydrogen atom from the pyridyl-type ring. The presence of a strongly deshielded signal at 9.25 ppm indicates strong nitrogen-hydrogen interaction, supporting curved benzo[h]quinolone architecture. These assignments are also in line with the analysis of remaining cross-peaks in 2D NOESY, ^{1}H-^{13}C HSQC and ^{1}H-^{13}C HMBC spectra.

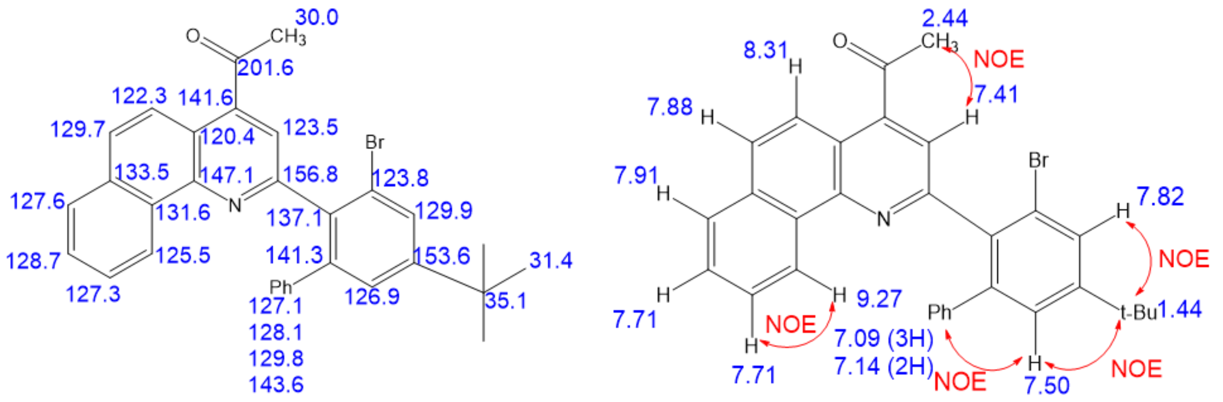

Figure S9. Full signal assignment for compound **4c**.

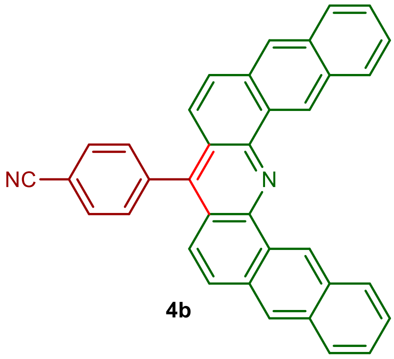

4b

High-resolution mass spectrometry determined the molecular formula of **4b** for 481.1711 peak as $C_{36}H_{21}N_2^+$ $[M+H]^+$. Structure proposed by the algorithm proved congruent with the spectroscopic data. Specifically, 1D and 2D NMR spectra evidence the presence of AA'XX' system originating from the parent aldehyde. Moreover, the presence of only 10 signals in the ^{1}H NMR spectra (integrated for 2 protons each) implies the molecule possesses C2 symmetry axis. Thus, remaining 29 carbon atoms probably belong to two anthracene-like moieties (2 x 14 C) bridged by the remaining carbon (originating from the formyl group) and nitrogen atoms (originating from amino group), in line with the proposed mechanism entailing condensation of one molecule of 4-cyanobenzaldehyde with two molecules of 1-anthrylamine (main-text **Figure 3a**). Judging from the multiplicity of the signals in the ^{1}H NMR spectra, as well as ^{1}H-^{1}H COSY correlations, protons were assigned to three spin systems: AA'XX' (7.94 and 7.62 ppm), ABCD (8.46, 8.12, 7.69 and 7.65 ppm), AB (7.76 and 7.24 ppm) with two sole singlets (10.32 and 8.39 ppm). The presence of ABCD and AB spin systems together with two sole singlets in the anthracene-like moiety (from the parent 1-antrylamine) indicates *a*-type fusion of the ring **III** with the central unit. Noteworthy, the latter should contain one nitrogen atom and 4-cyanophenyl substituent *para-* to each other, to secure the C2 symmetry of the molecule. Remaining cross-peaks in 2D NOESY, ^{1}H-^{13}C HSQC and ^{1}H-^{13}C HMBC spectra are also in line with this reasoning. Interestingly, the increased intensity of the signal at 132.4 ppm at the ^{13}C NMR spectra indicates it is the combination of two peaks (belonging to the side substituent and quaternary carbon from the terminal six-membered ring **I**), explaining artificial extremely long-range (through 7 bonds) at the ^{1}H-^{13}C HMBC correlation {10.32, 132.4}.

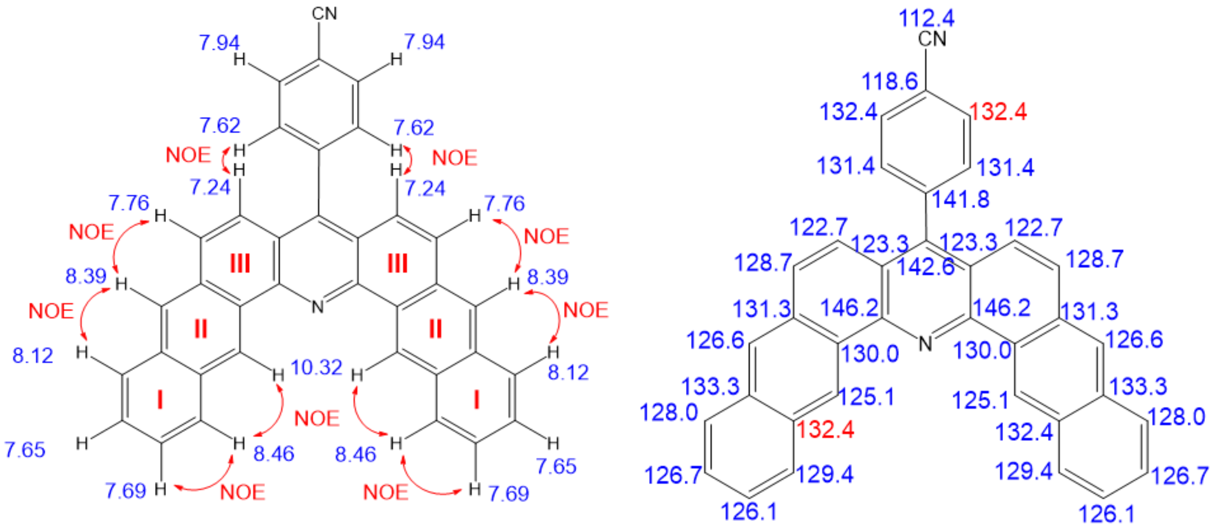

Figure S10. Full signal assignment for compound **4b**.

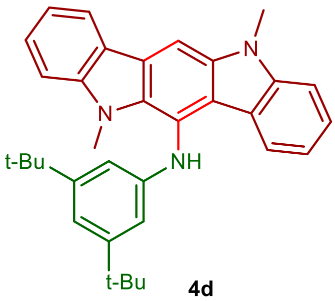

4d

High-resolution mass spectrometry determined the molecular formula of **4d** for 487.2992 peak as $C_{34}H_{37}N_3$. Again, spectral assignments were guided by and congruent with the mechanism predicted by the algorithm (main-text **Figure 3c**). Specifically, the upfield singlets in ^{1}H NMR [4.00 ppm (3H, CH_3), 3.95 ppm (3H, CH_3) and 1.15 ppm (18H, 2 x *t*-Bu)] are indicative of the proposed structure. Based on correlations from the ^{1}H-^{1}H COSY experiment, 13 protons from the aromatic region can be assigned to 3 spin systems [4H (ABCD) + 4H (A'B'C'D') +3H (A_2B)] along with two sole signals at 8.05 and 7.27 ppm. Even though the analysis of the ^{1}H-^{1}H COSY spectra is challenging due to the merging signals (7.40-7.37 ppm, multiplet for 3H), the ^{1}H-^{13}C HMBC cross-peaks clearly identified them as belonging to two different spin systems (correlations {8.18, 126.4} and {7.14, 109.1} from the first as well as {6.96, 108.2 ppm} from the second one). Thus, final assignment of protons originating from parent aldehyde is as follows: ABCD spin system (8.18 ppm, 7.14 ppm, two protons from the multiplet at 7.40-7.37 ppm) and A'B'C'D' spin system (8.09 ppm, 7.33 ppm, 6.96 ppm, one from the multiplet at 7.40-7.37 ppm). Noteworthy, the 2D NOESY experiment shows cross-relaxation between singlet at 1.15 ppm (2 x *t*-Bu) and signals at 6.63 ppm as well as 6.79 ppm, belonging to the A_2B spin system. Moreover, through-space interaction between peaks at 6.63 ppm (from the A_2B system) and 7.27 ppm (sole singlet, integration suggests an exchangeable proton) indicates that the A_2B spin system belongs to substituent originating from aniline. In addition, the integration of the exchangeable proton implies the substituent is attached to the rest of the molecule through the single C-N bond.

Structural assignment of the residual part turned out to be more demanding. The $C_{34}H_{37}N_3$ formula suggests the units originating from parent indole are linked through two carbons arising from the formyl groups forming inner 6-membered ring. The initial indication came from the 2D NOESY experiment, which shows through-space interaction between isolated proton at 8.05 ppm and “terminal” proton from the ABCD spin system {8.18, 8.05} as well as one methyl group {3.95, 8.05}. Moreover, the cross-peaks of the signal at 4.00 ppm (second methyl group) with exchangeable NH proton (7.27 ppm) as well as with broad singlet from A_2B spin system

(substituent originating from aniline) and "terminal" proton from the A'B'C'D' spin system support the structure of dihydroindolo[3,2-*b*]carbazole (**Figure S11**).These assignments are in line with the remaining cross-peaks in 2D NOESY, ^{1}H-^{13}C HSQC and ^{1}H-^{13}C HMBC spectra which fully supports previous considerations. In addition, all ^{1}H NMR chemical shifts are congruent with data previously reported for similar indolo[3,2-*b*]carbazoles.[S2]

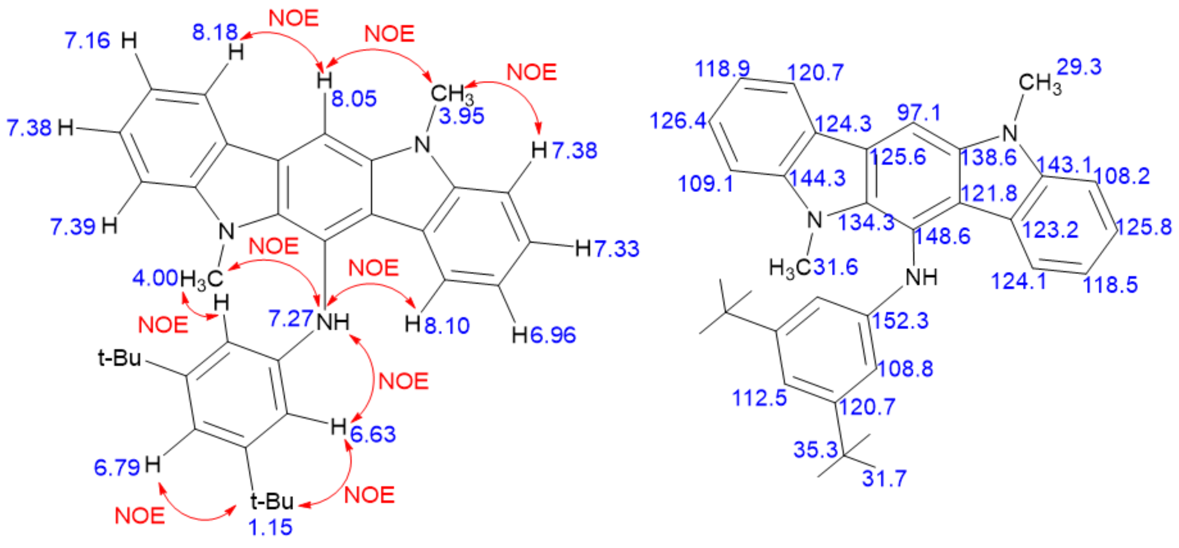

Figure S11. Full signal assignment for compound **4d**.

Section S3.4. 1H and 13C NMR spectra for synthesized compounds

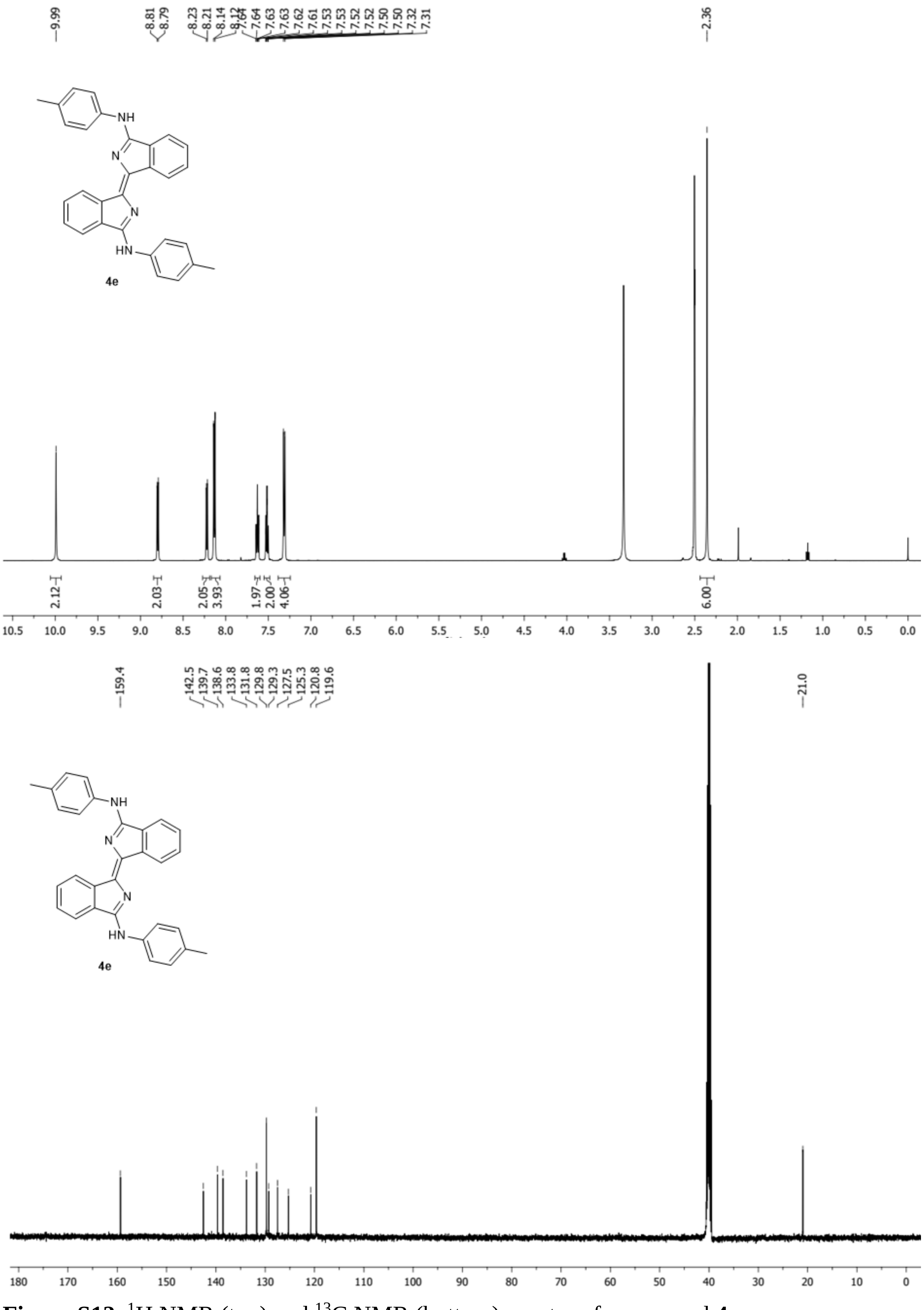

Figure S12. ^{1}H NMR (top) and ^{13}C NMR (bottom) spectra of compound **4e**.

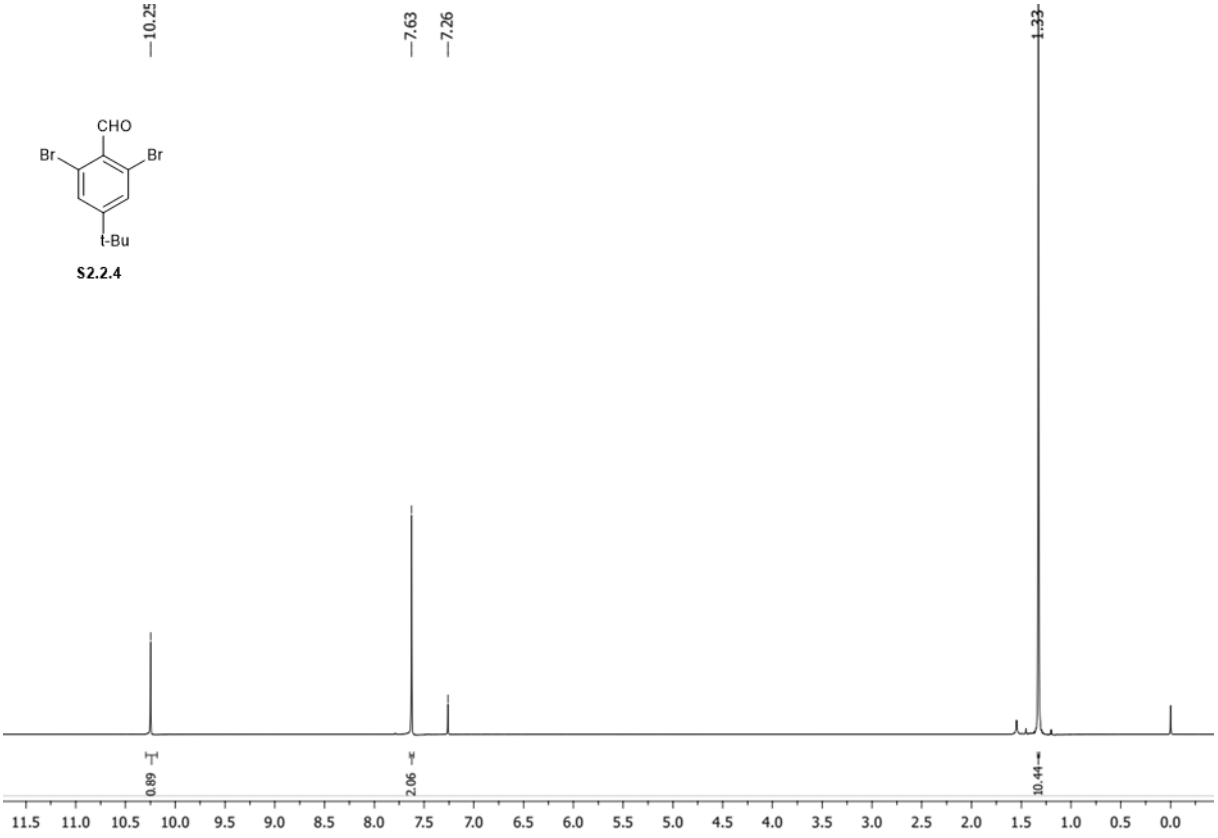

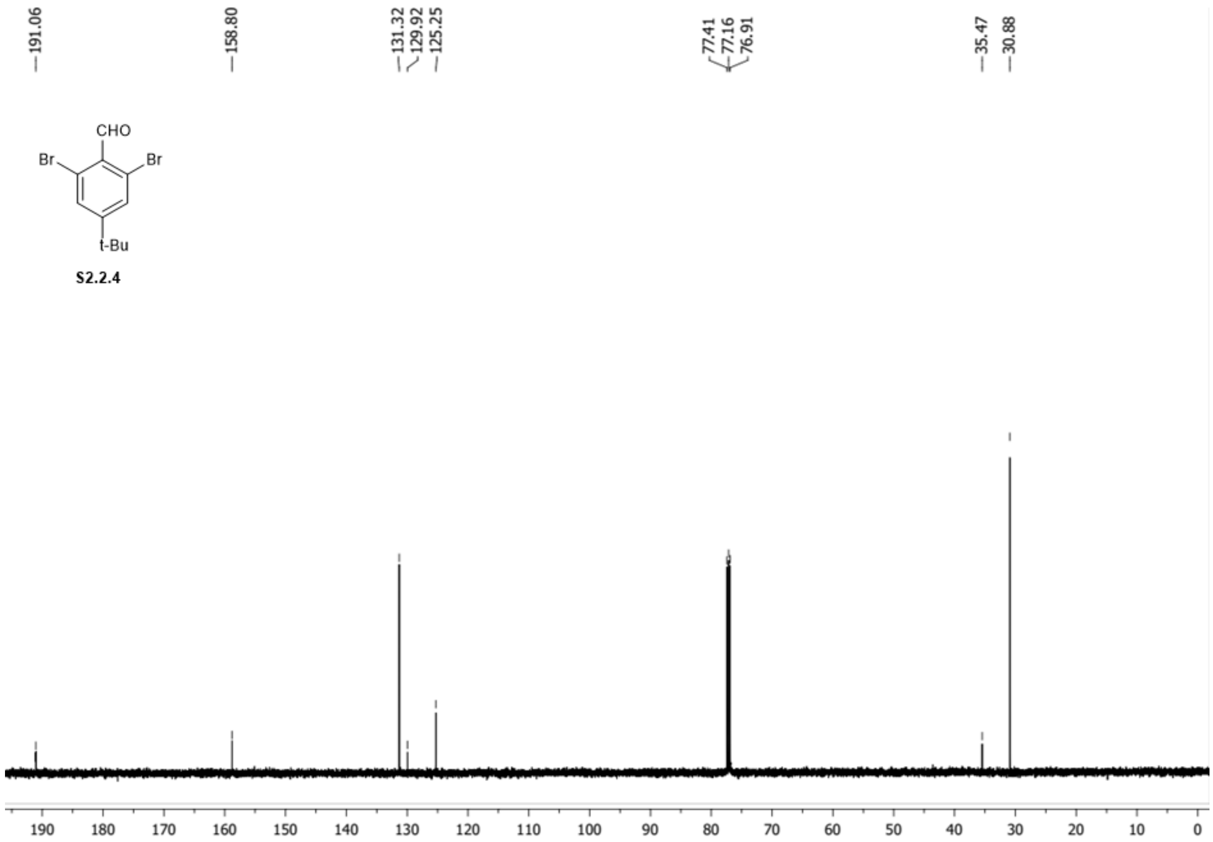

Figure S13. ^{1}H NMR (top) and ^{13}C NMR (bottom) spectra of compound **S2.2.4**.

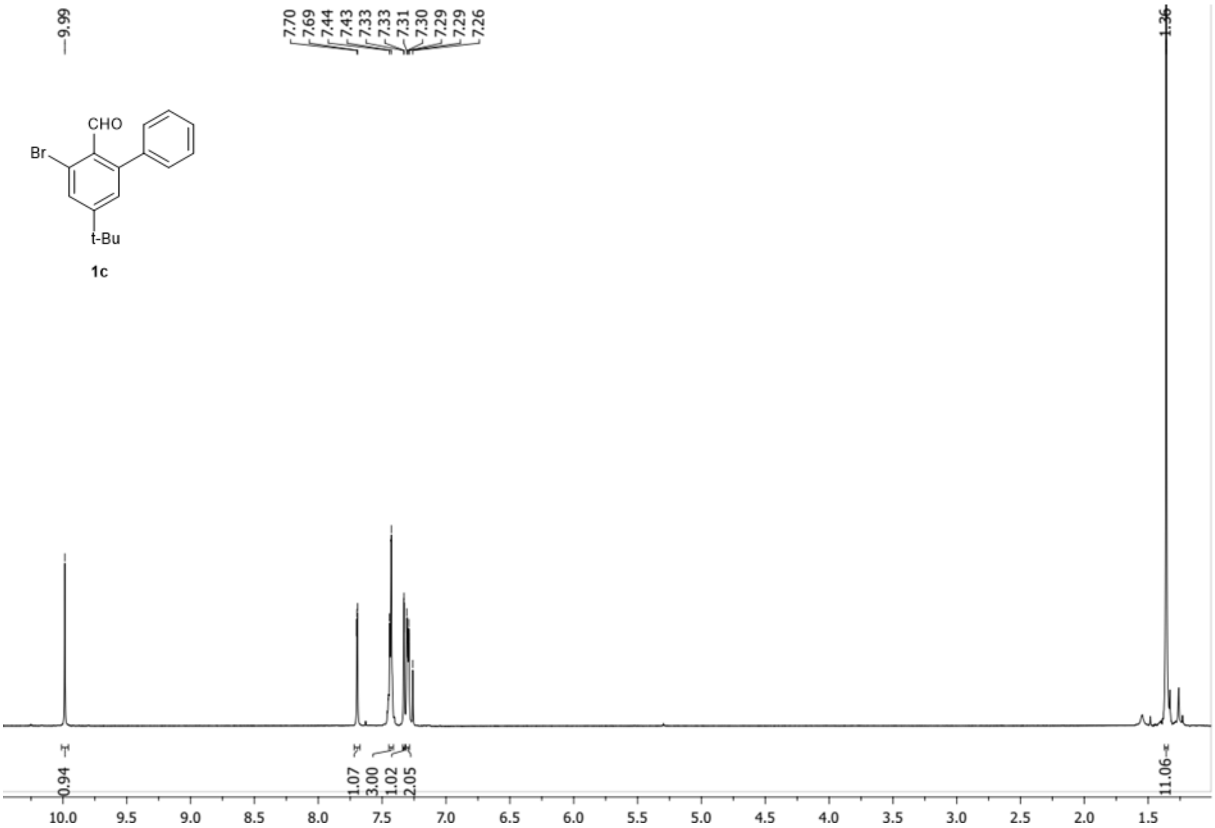

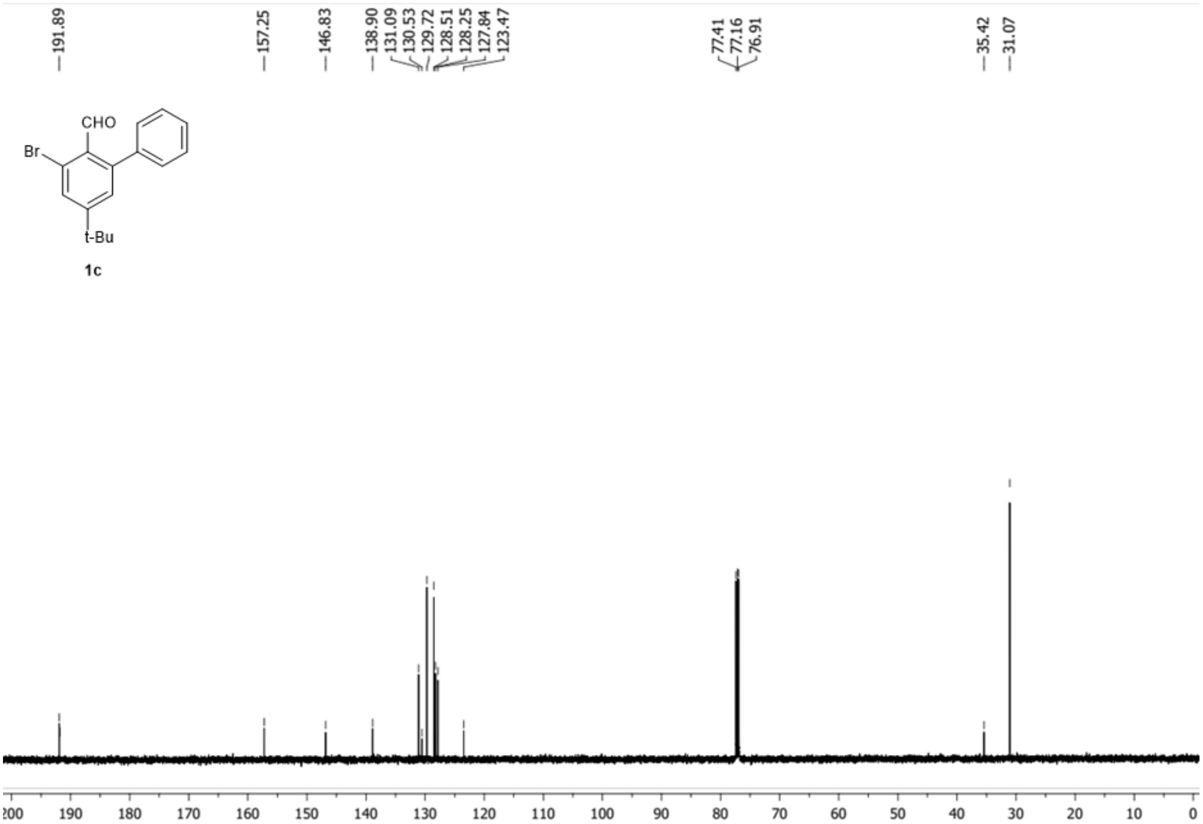

Figure S14. ^{1}H NMR (top) and ^{13}C NMR (bottom) spectra of compound **1c**.

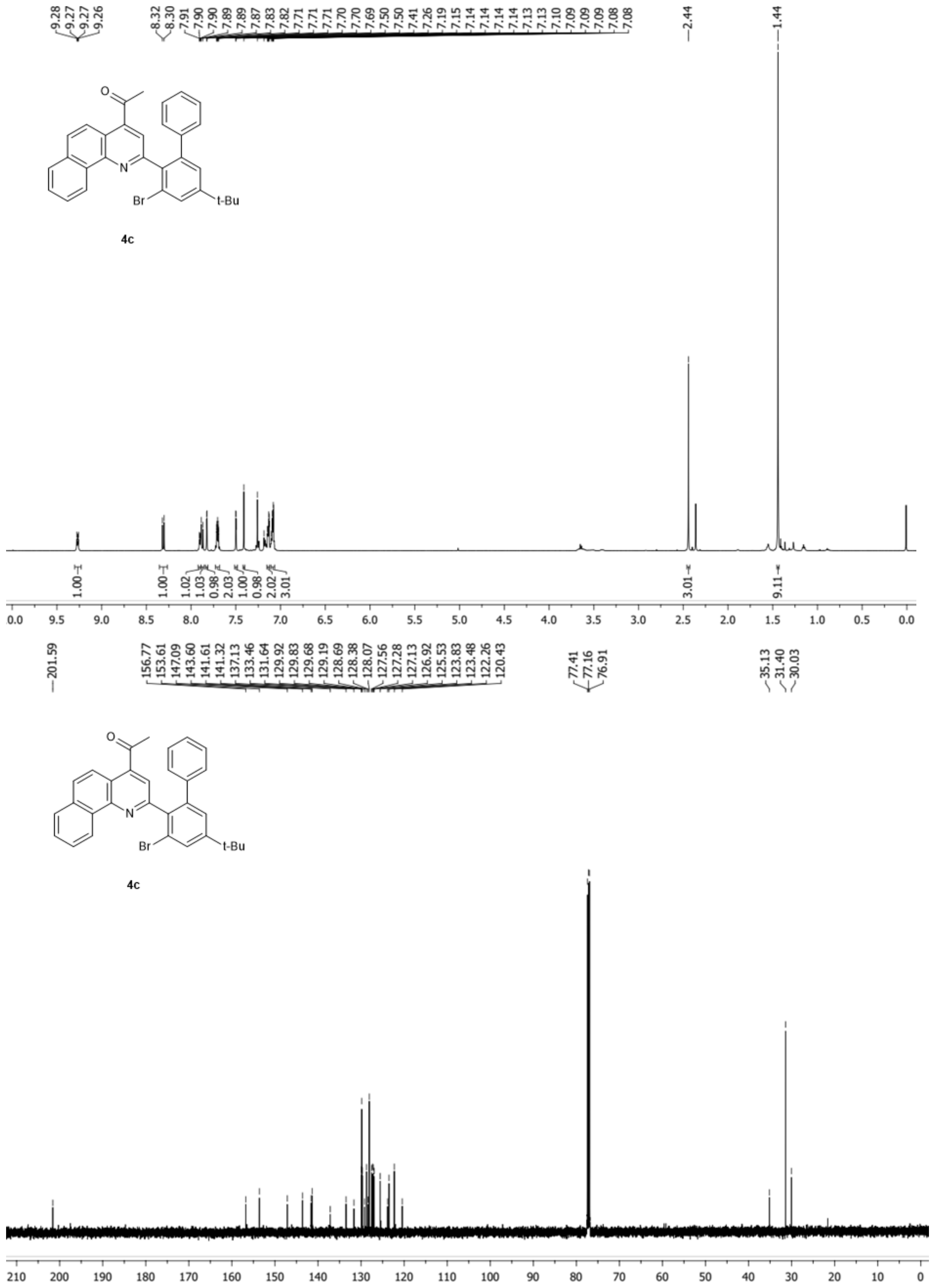

Figure S15. ^{1}H NMR (top) and ^{13}C NMR (bottom) spectra of compound **4c**.

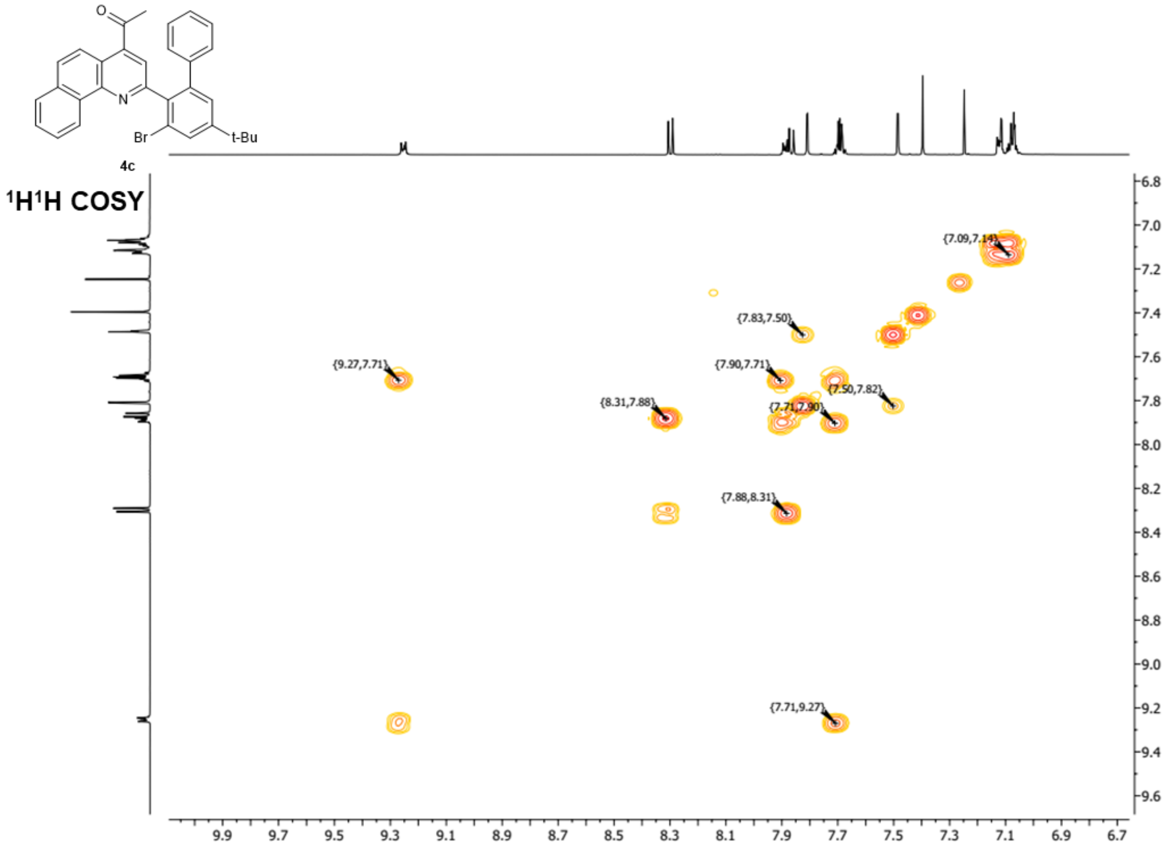

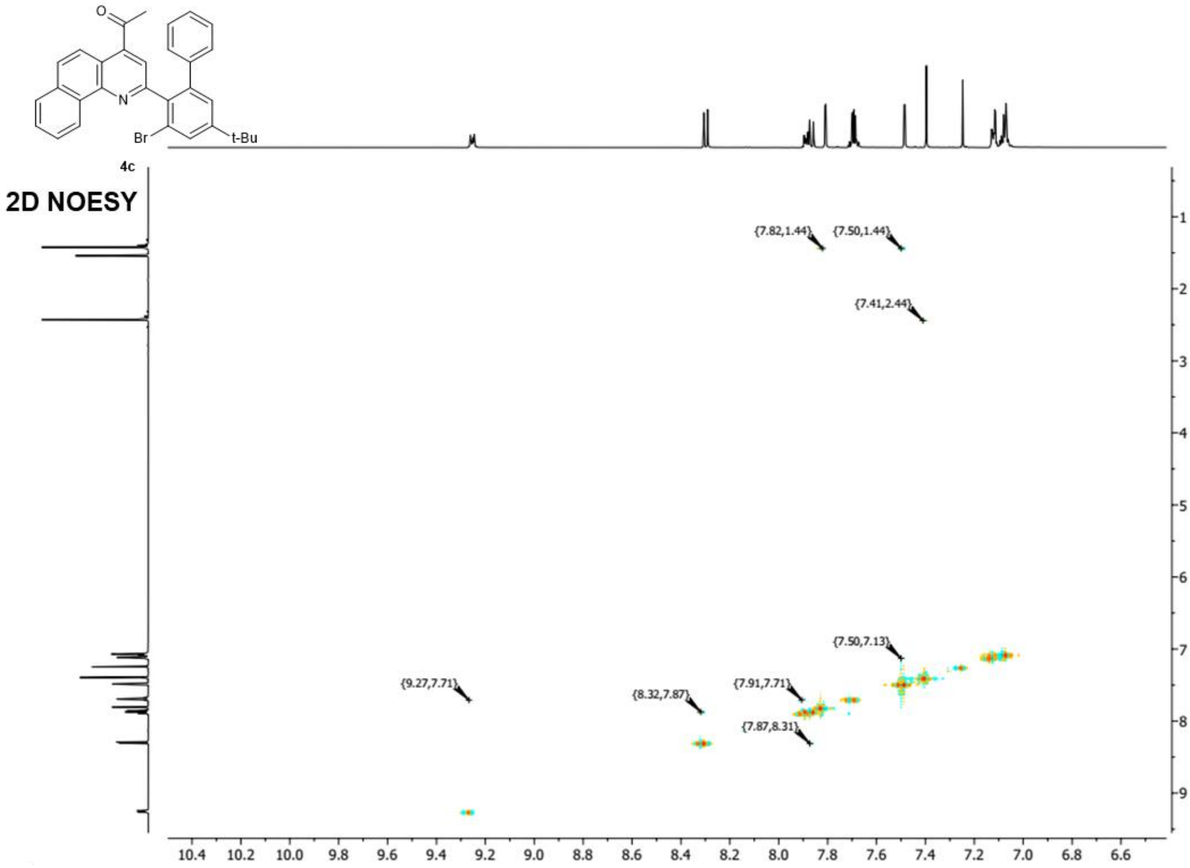

Figure S16. $^{1}H^{1}H$ COSY (top) and 2D NOESY (bottom) spectra of compound **4c**.

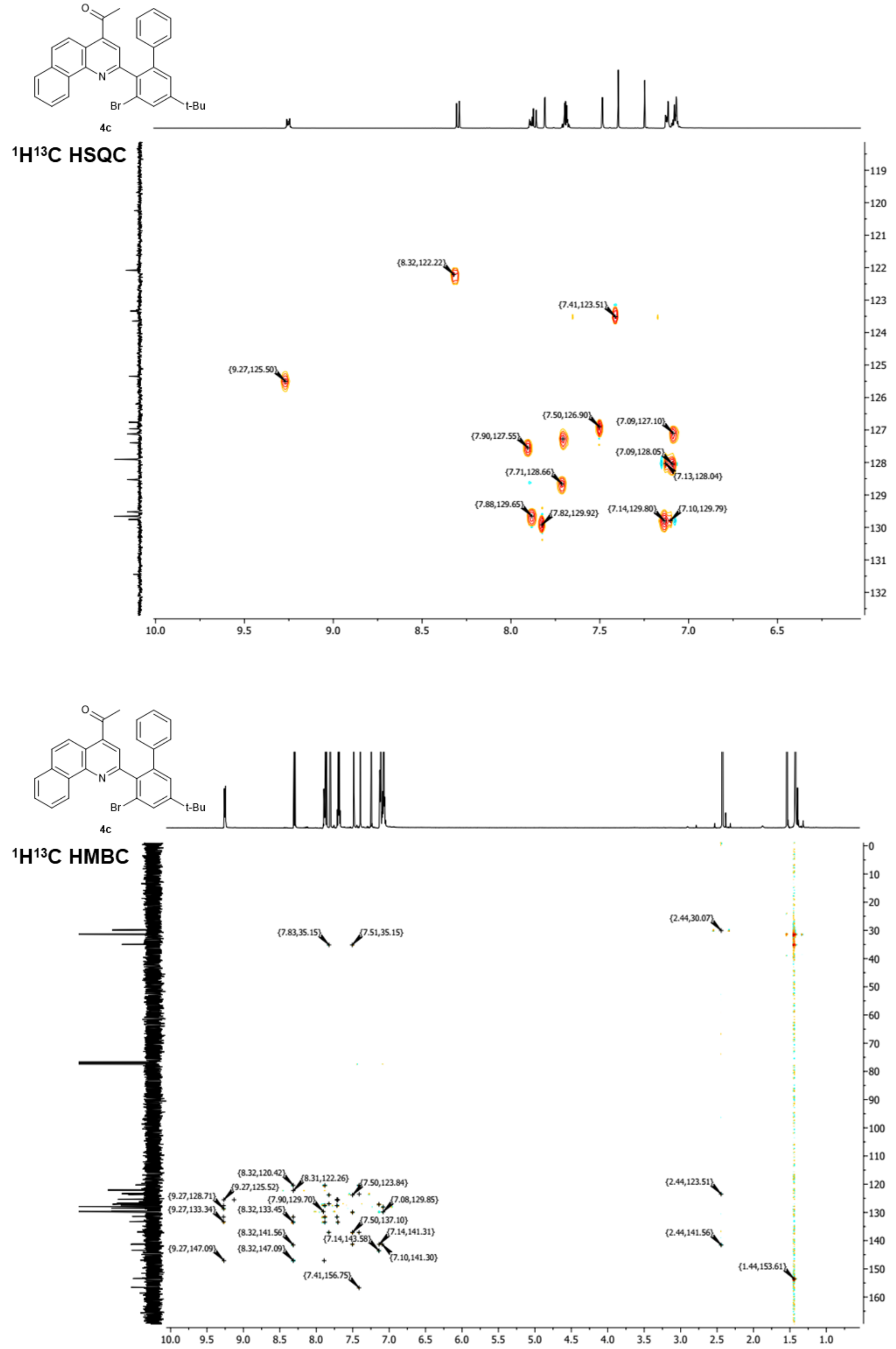

Figure S17. $^{1}H^{13}C$ HSQC (top) and $^{1}H^{13}C$ HMBC (bottom) spectra of compound **4c**.

Single Mass Analysis

Tolerance = 5.0 mDa / DBE: min = -1.5, max = 300.0

Element prediction: Off

Number of isotope peaks used for i-FIT = 3

Monoisotopic Mass, Even Electron Ions

66 formula(e) evaluated with 1 results within limits (up to 50 closest results for each mass)

Elements Used:

C: 0-120 H: 0-200 N: 1-2 Br: 0-2

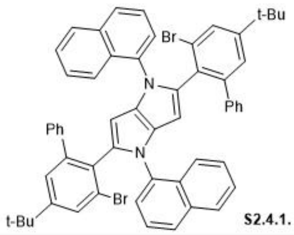

S2.4.1.

Mass	Calc. Mass	mDa	PPM	DBE	Formula	i-FIT	i-FIT Norm	Fit Conf %	C	H	N	Br
931.2256	931.2263	-0.7	-0.8	34.5	C58 H49 N2 Br2	494.7	n/a	n/a	58	49	2	2

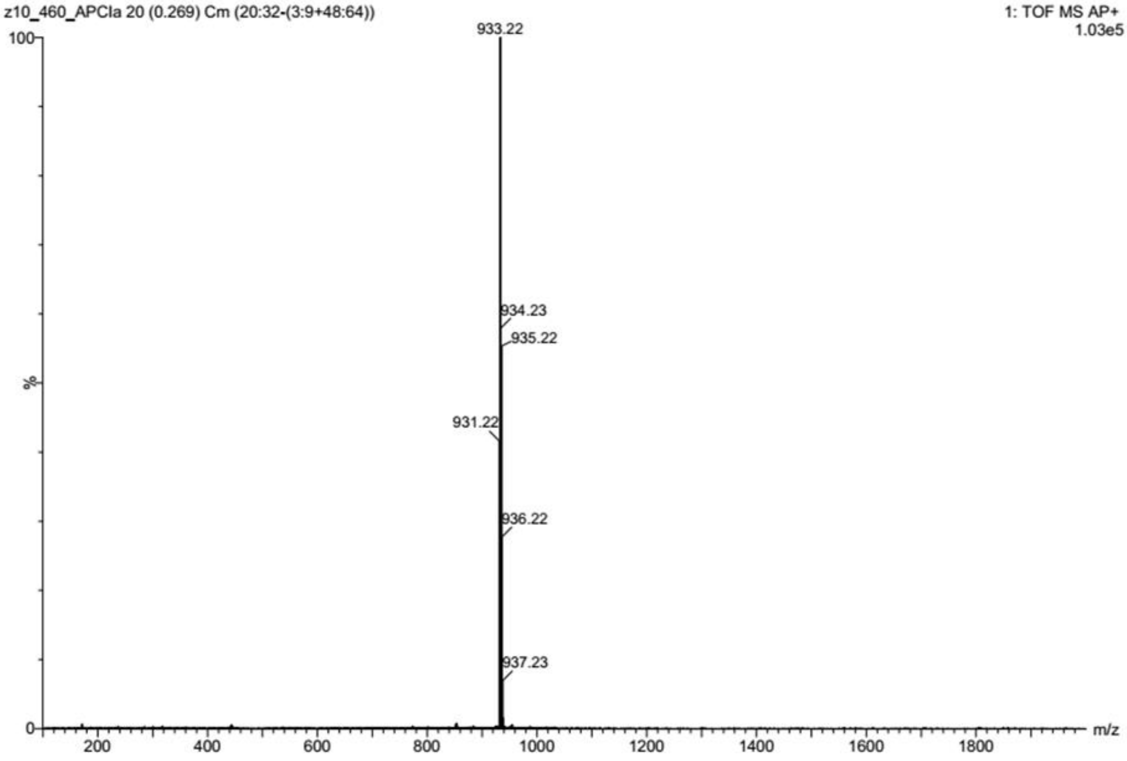

Figure S18. MS spectra of **S2.4.1.**

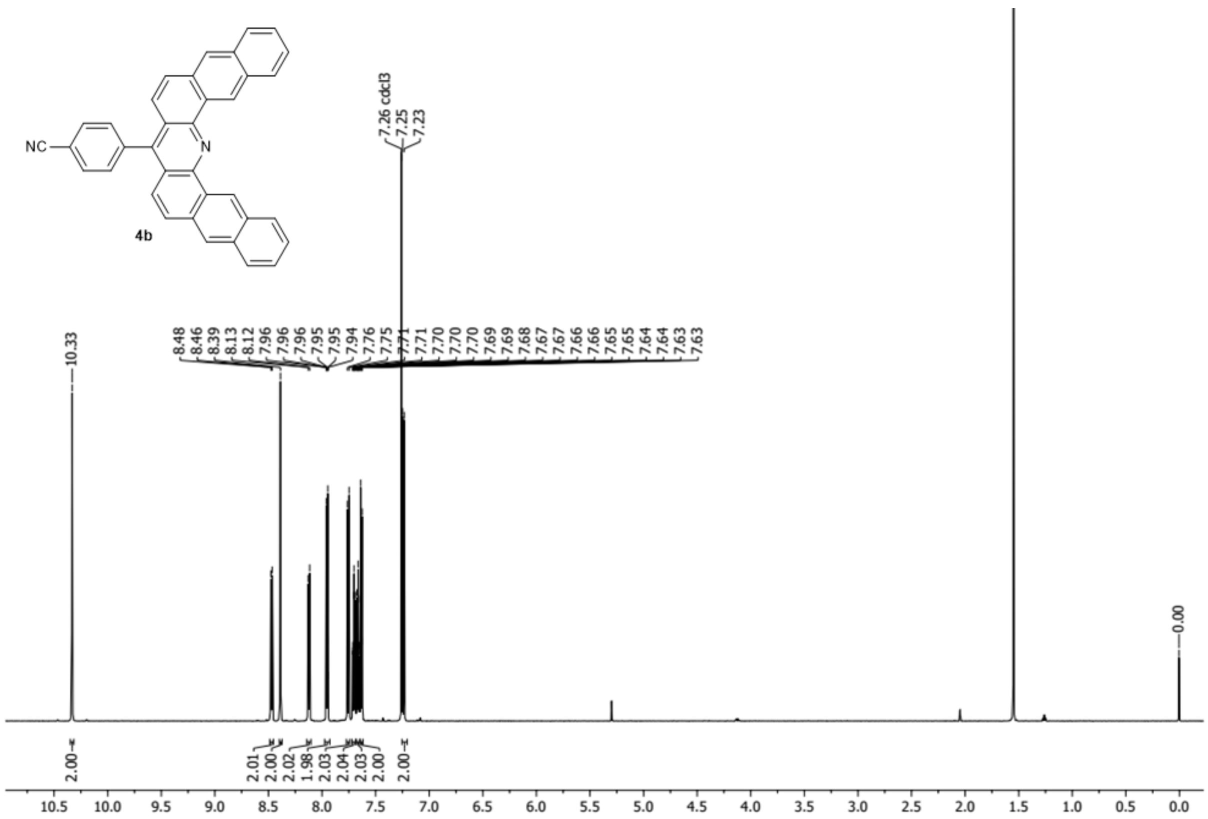

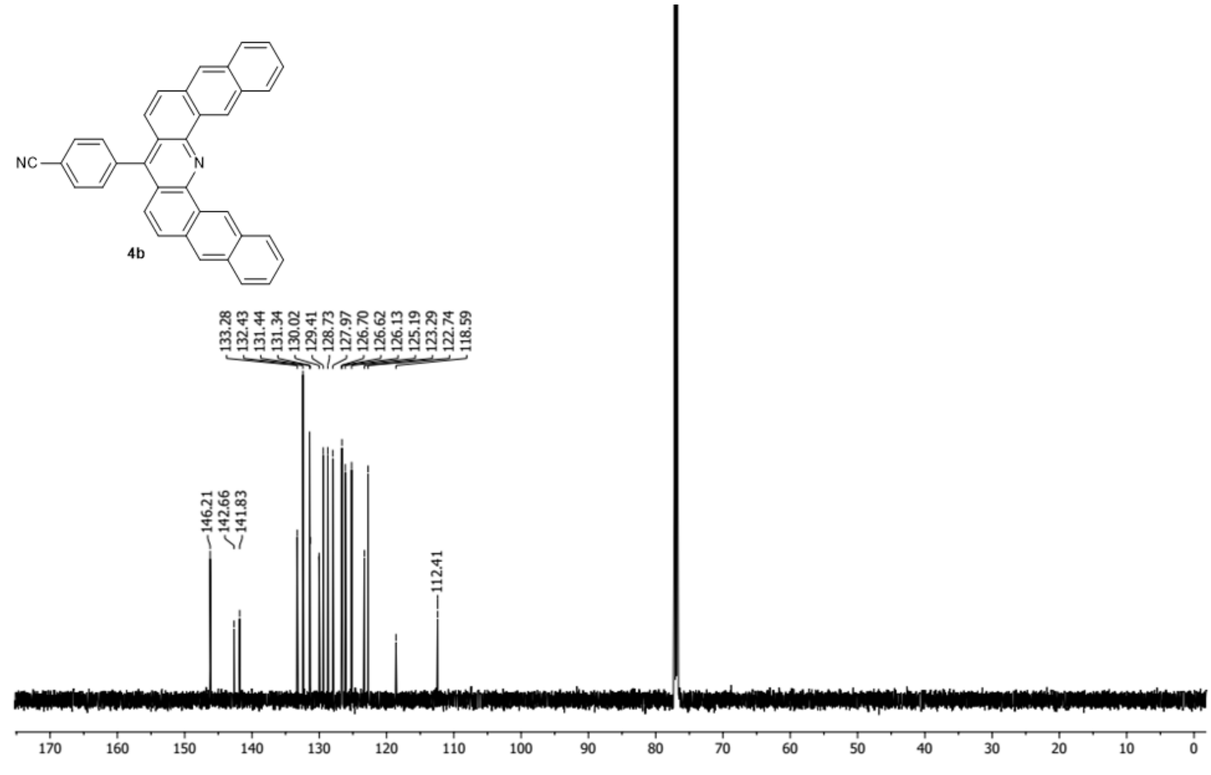

Figure S19. ^{1}H NMR (top) and ^{13}C NMR (bottom) spectra of compound **4b**.

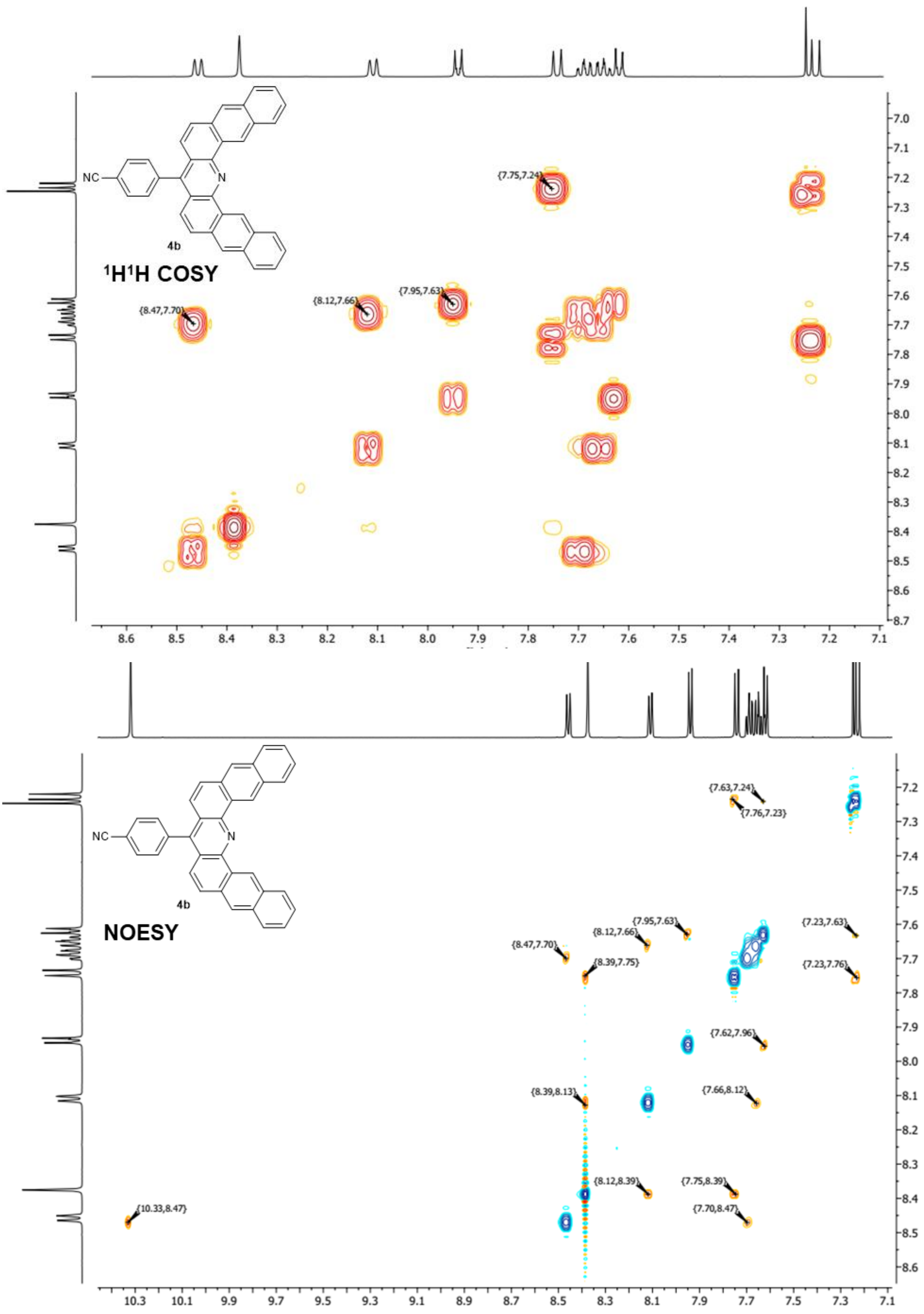

Figure S20. ^{1}H^{1}H COSY (top) and 2D NOESY (bottom) spectra of compound **4b**.

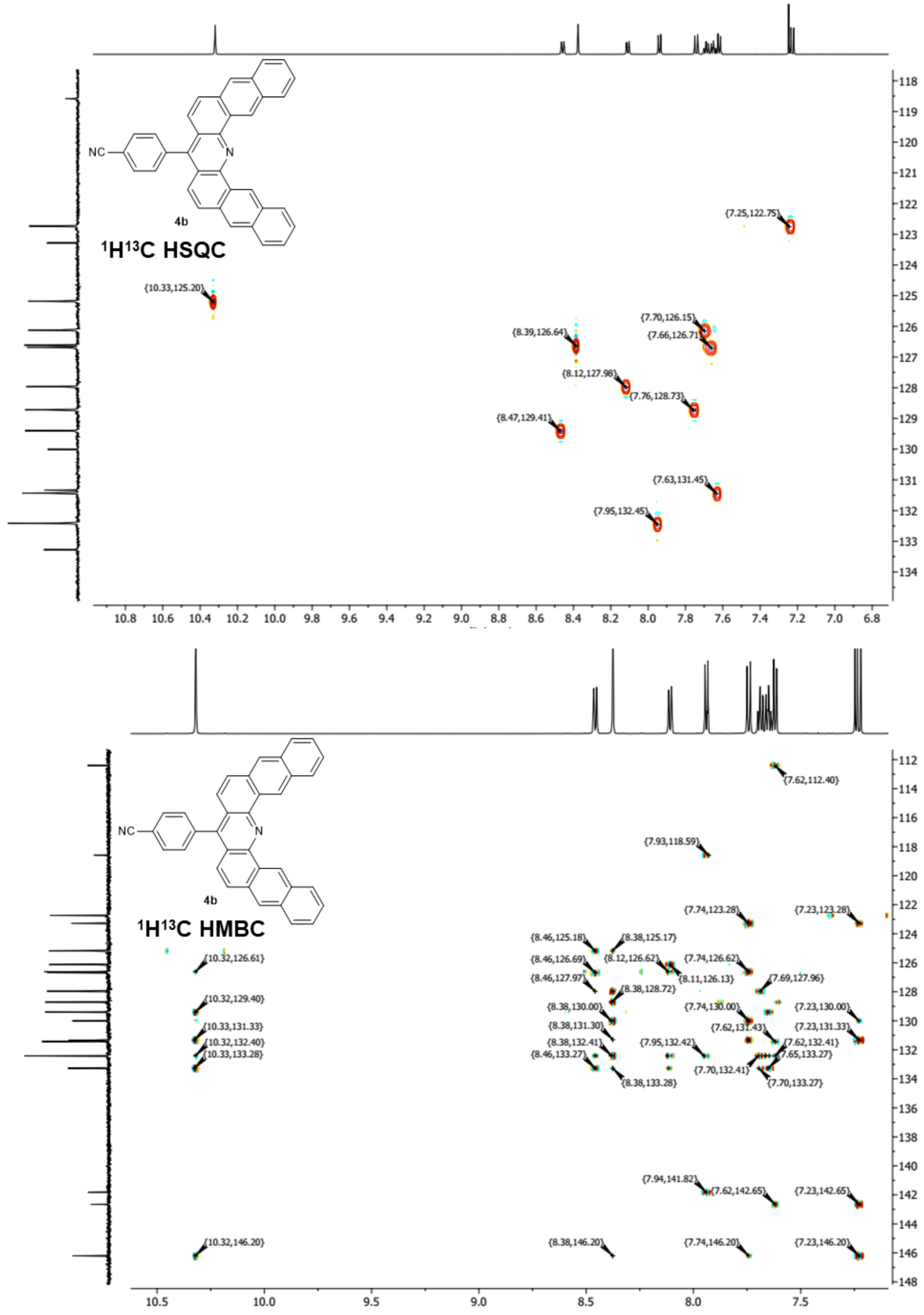

Figure S21. $^{1}H^{13}C$ HSQC (top) and $^{1}H^{13}C$ HMBC (bottom) spectra of compound **4b**.

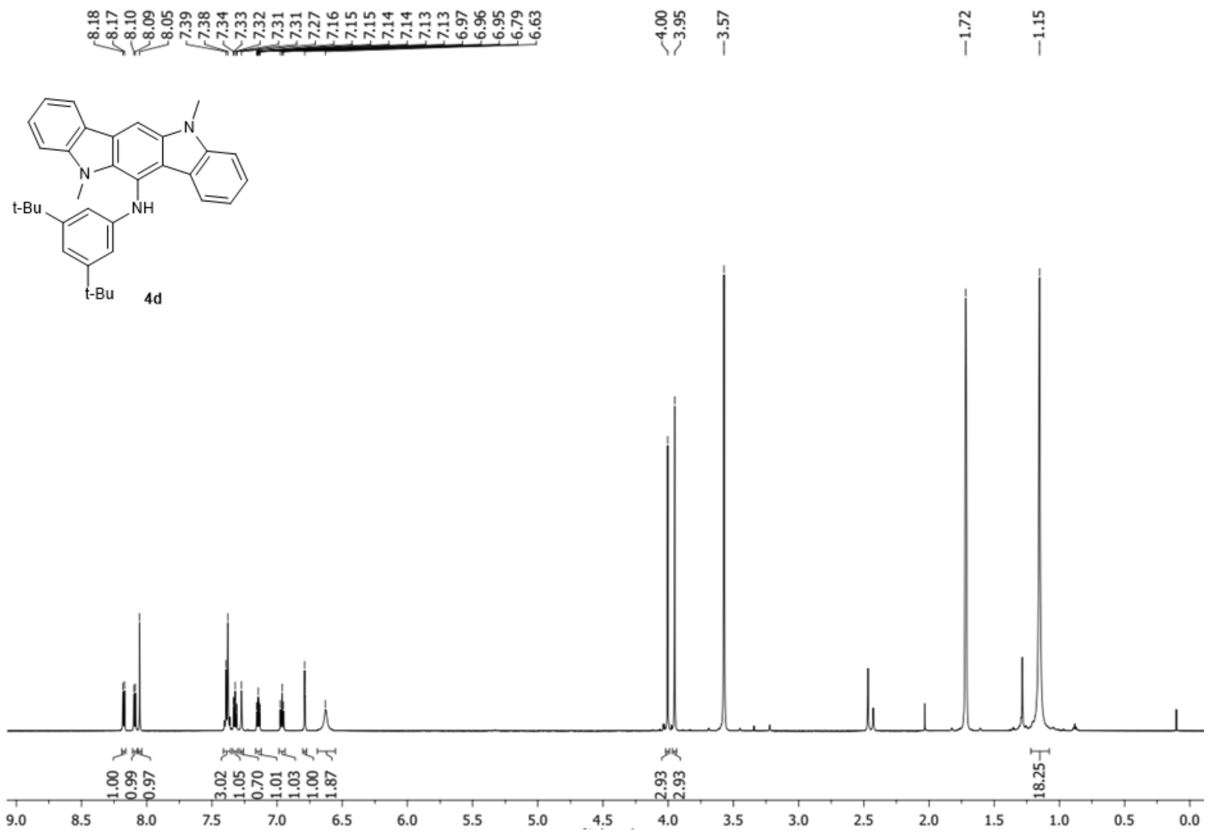

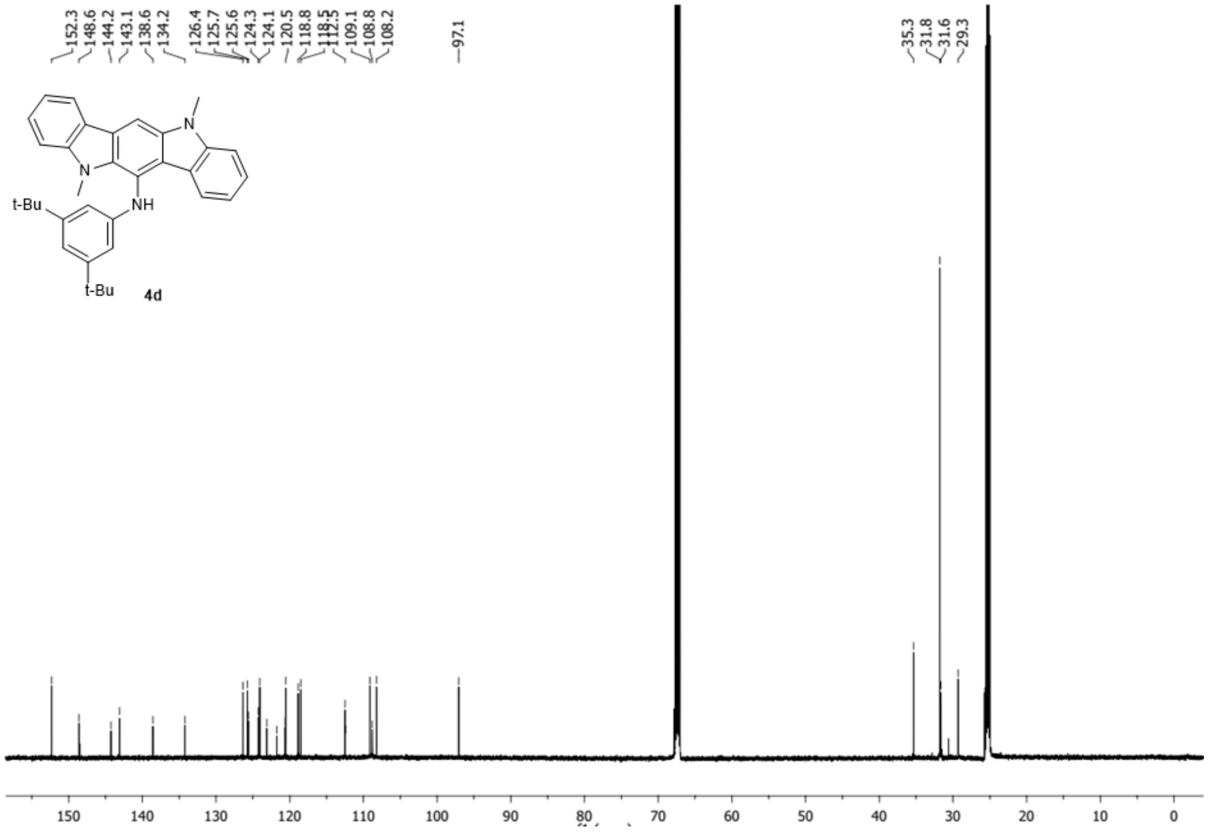

Figure S22. ^{1}H NMR (top) and ^{13}C NMR (bottom) spectra of compound **4d**.

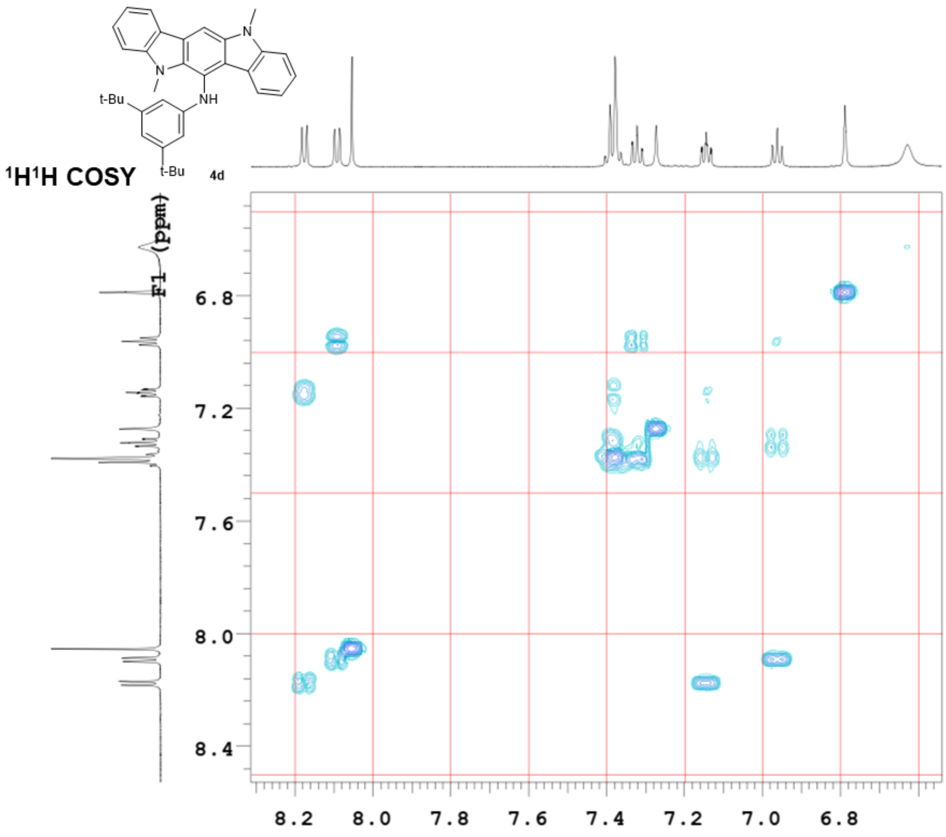

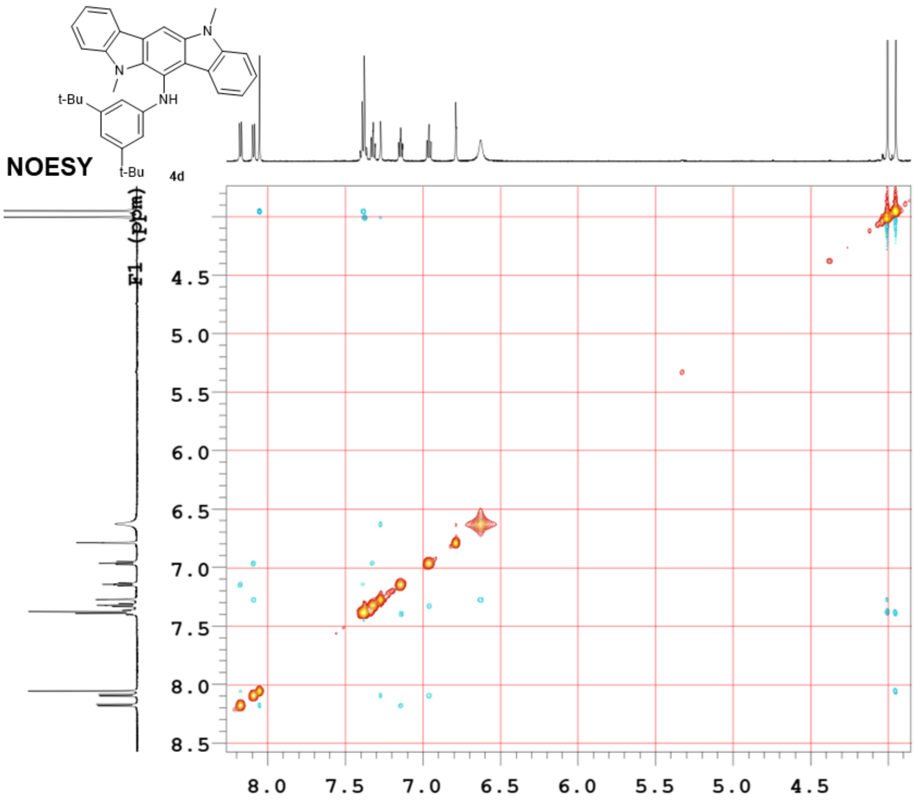

Figure S23. $^{1}H^{1}H$ COSY (top) and 2D NOESY (bottom) spectra of compound **4d**.

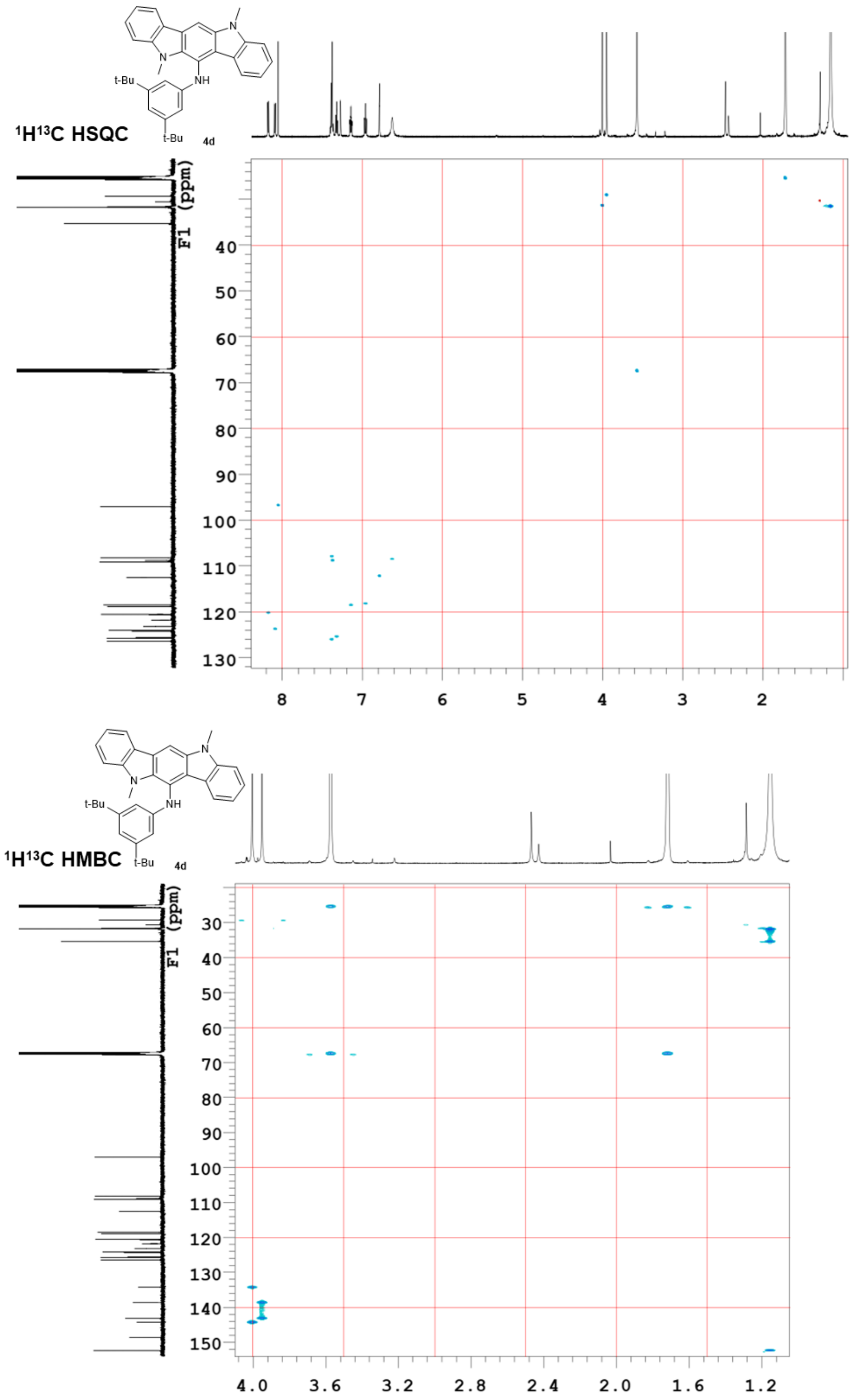

Figure S24. $^1H^{13}C$ HSQC (top) and $^1H^{13}C$ HMBC (bottom) spectra of compound **4d**.

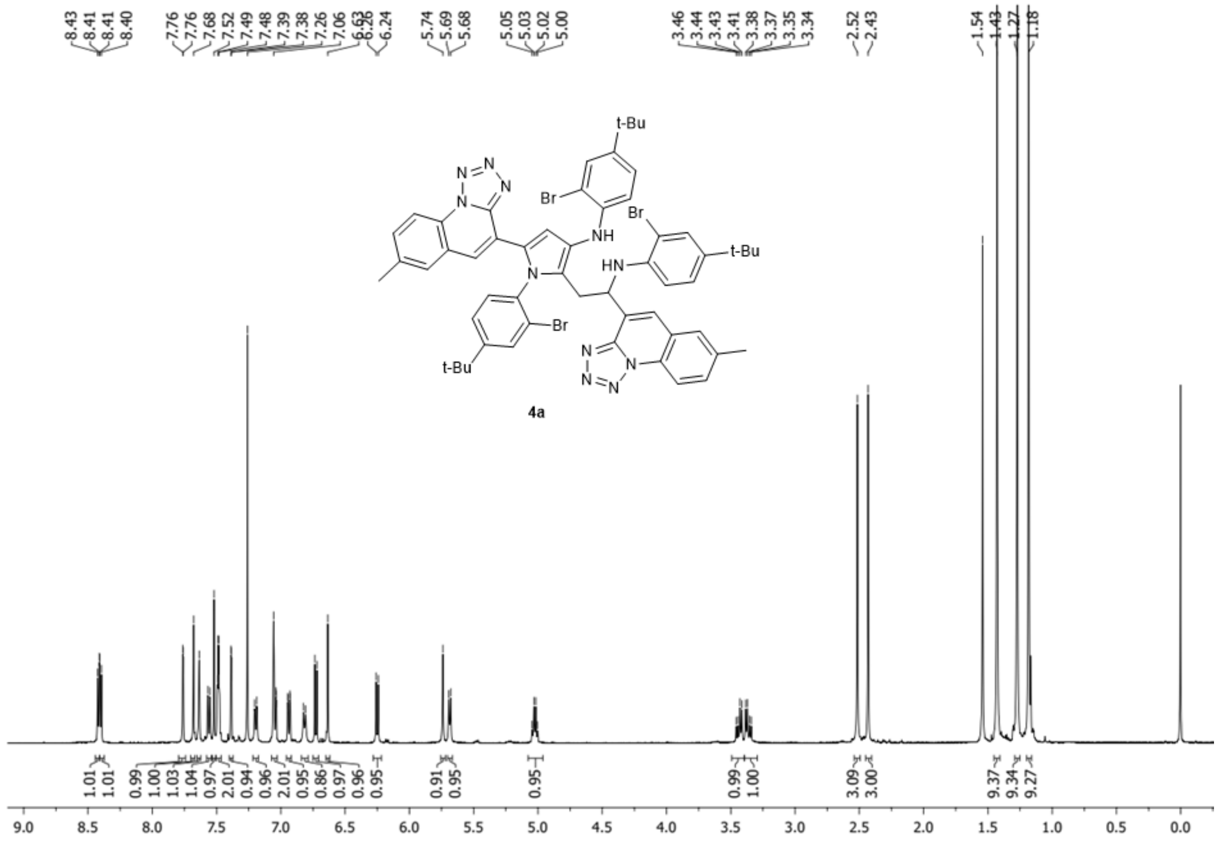

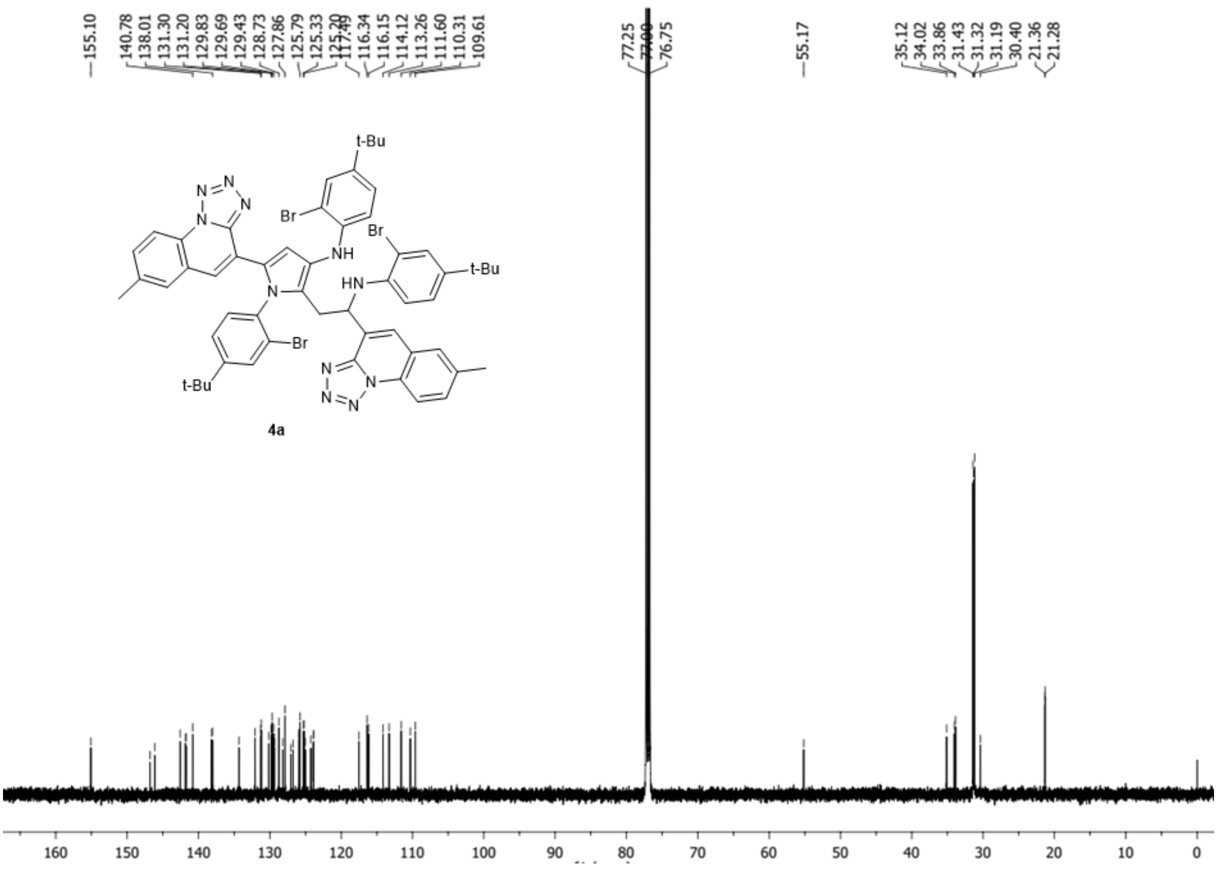

Figure S25. ^{1}H NMR (top) and ^{13}C NMR (bottom) spectra of compound **4a**.

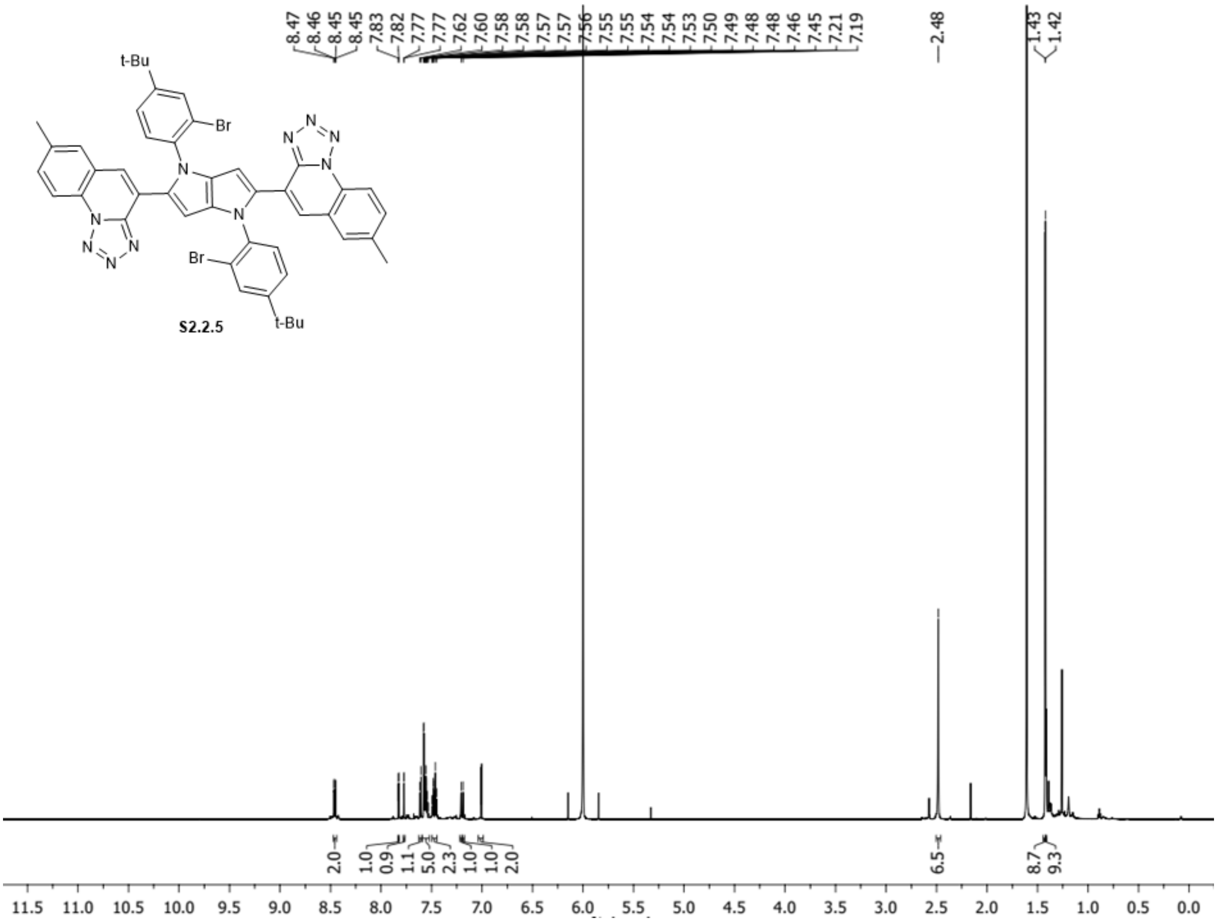

Figure S26. ^{1}H NMR spectra of compound **S2.2.5**.

Section S3.5. Crystallographic data for 4a and 4e.

Crystallographic data were collected on a Bruker X8 APEXII diffractometer with Cu-Kα radiation (λ = 1.54178 Å). Frames were integrated with the Bruker SAINT[S3] software package using a narrow-frame algorithm. The structures ware solved and refined using the Bruker SHELXTL Software Package.[S4,S5] All obtained data were corrected for absorption effects using the face-indexed numerical method (SA-DABS).[S6] Same hydrogens were found from the difference electron density maps and refined with an anisotropic thermal motion model. Other hydrogen atoms were placed in calculated positions and refined as riding on their parent atoms with Uiso = 1.2 Ueq. The structure was solved by direct methods SHELXS-2014 and refined with full-matrix least-squares calculations on F2 using SHELX-2014.[S7] All non-hydrogen atoms were refined anisotropically.

Crystallographic data for compound 4a (CCDC Number: 2320965**)**

Single crystal of **4a** suitable for X-ray diffraction analysis was obtained by slowly diffusing acetonitrile into its chloroform solution.

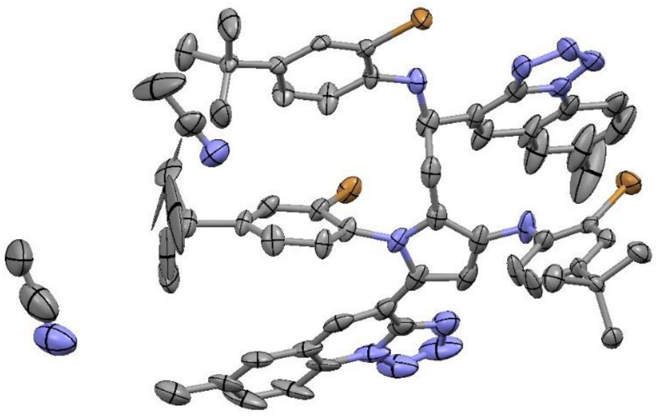

Figure S27. Crystal structure of **4a** with thermal ellipsoids, shown at 50% probability. The hydrogen atoms in the structure were omitted for clarity

Table S1. Crystallographic data for **4a**

Chemical Formula	$C_{60}H_{62}Br_3N_{13}$

Formula weight	1204.95 g/mol	
Temperature	150(2) K	
Wavelength	1.54178 Å	
Crystal size	0.165×0.194×0.311	
Crystal habit	pale-yellow needle	
Crystal system	triclinic	
Space group	$P\bar{1}$	
Unit cell dimensions	a = 9.6275(2) Å	α = 87.929(2)°
	b = 15.5602(3) Å	β = 84.8800(10)°
	c = 19.0439(4) Å	γ = 80.1630(10)°
Volume	2799.09(10) $Å^3$	
Z	2	
Diffractometer	Bruker APEX-II CCD	
Radiation source	fine-focus sealed tube	
Reflections collected	63742	
Independent reflections	9101 [R(int) = 0.1182]	
Tmax, Tmin	0.6310 and 0.4480	
Absorption correction	Numerical (SADABS)	
Refinement method	Full-matrix least-squares on F^2	

Function minimized	Σ w(F_o^2 - F_c^2)^2	
Data/restraints/parameters	9101 / 8 / 701	
Goodness-of-fit on F2	1.023	
Final R indices	4404 data; I>2σ(I)	R1 = 0.0919, wR2 = 0.1227
	all data	R1 = 0.2072, wR2 = 0.1528
Largest diff. Peak and hole	0.794 and -0.748 eÅ^{-3}	

Crystallographic data for compound 4e (CCDC Number: 2313809**)**

Single crystal of **4e** suitable for X-ray diffraction analysis was obtained by slowly diffusing hexanes into its chloroform solution. A total of 4367 frames were collected. The total exposure time was 41.24 hours.

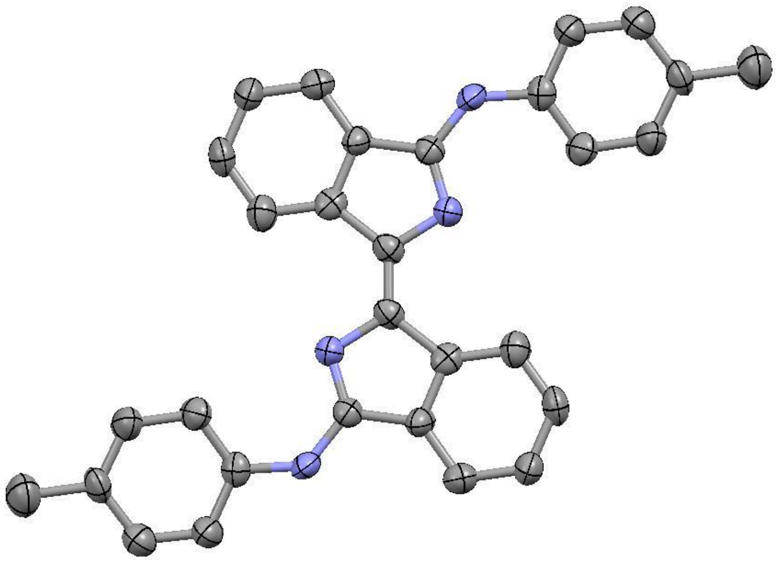

Figure S28. Crystal structure of **4e** with thermal ellipsoids, shown at 50% probability. The hydrogen atoms in the structure were omitted for clarity

Table S2. Crystallographic data for **4e.**

Chemical Formula	$C_{30}H_{24}N_4$
Formula weight	440.5 g/mol
Temperature	296(2) K
Wavelength	1.54178 Å
Crystal size	0.111 x 0.146 x 0.445 mm
Crystal habit	red needle
Crystal system	monoclinic

Space group	P1 2_1/c 1	
Unit cell dimensions	a = 13.199(4) Å	α = 90°
	b = 6.0521(18) Å	β = 113.353(19)°
	c = 15.046(4) Å	γ = 90°
Volume	1103.4(6) $Å^3$	
Z	2	
Diffractometer	Bruker APEX-II CCD	
Radiation source	fine-focus sealed tube	
Reflections collected	9255	
Independent reflections	1467 [R(int) = 0.0776]	
Tmax, Tmin	0.9350, 0.7710	
Absorption correction	Numerical (SADABS)	
Refinement method	Full-matrix least-squares on F^2	
Function minimized	Σ w(F_o^2 - F_c^2)^2	
Data/restraints/parameters	1467 / 0 / 187	
Goodness-of-fit on F2	1.239	
Final R indices	990 data; I>2σ(I)	R1 = 0.1217, wR2 = 0.2791
	all data	R1 = 0.1819, wR2 = 0.2984

Largest diff. Peak and hole	0.281 and -0.312 eÅ^{-3}

Section S3.6. Optical properties of compounds 4a-4e (in toluene).

The absorbance and fluorescence spectra were measured on Perkin–Elmer Lambda 25 UV/VIS and Hitachi F-7000 respectively. Linear optical measurements Steady-state fluorescence measurements were performed with dilute solutions (10^{-6} M, optical density < 0.1) contained in standard 1 cm quartz cuvettes at room temperature. Compounds were dissolved in toluene unless otherwise noted. Fluorescence quantum yields were measured by using Coumarin 153 in ethanol (0.55) as a standard.

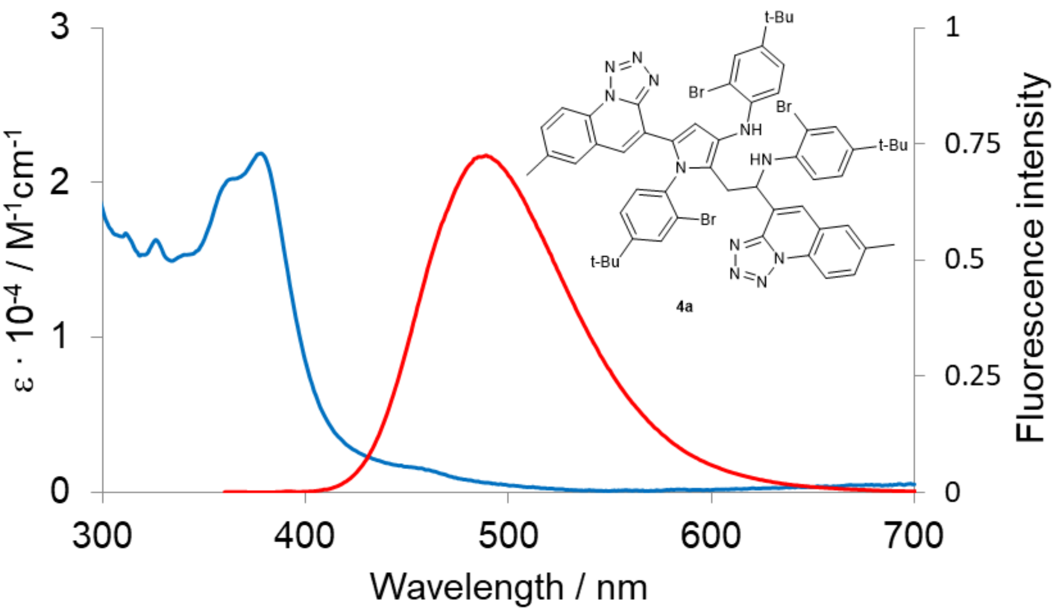

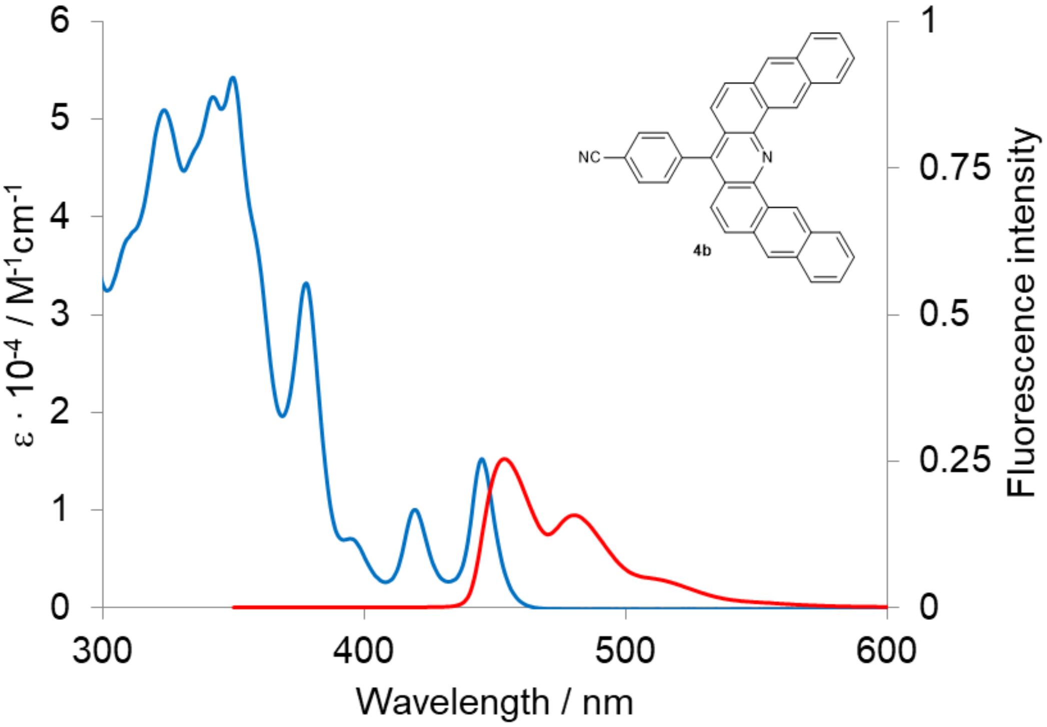
6
5
4
3
2
1
0
ε · 10-4 / M-1cm-1
1
0.75
0.5
0.25
0
Fluorescence intensity
300
400
500
600
Wavelength / nm
NC
N
4b

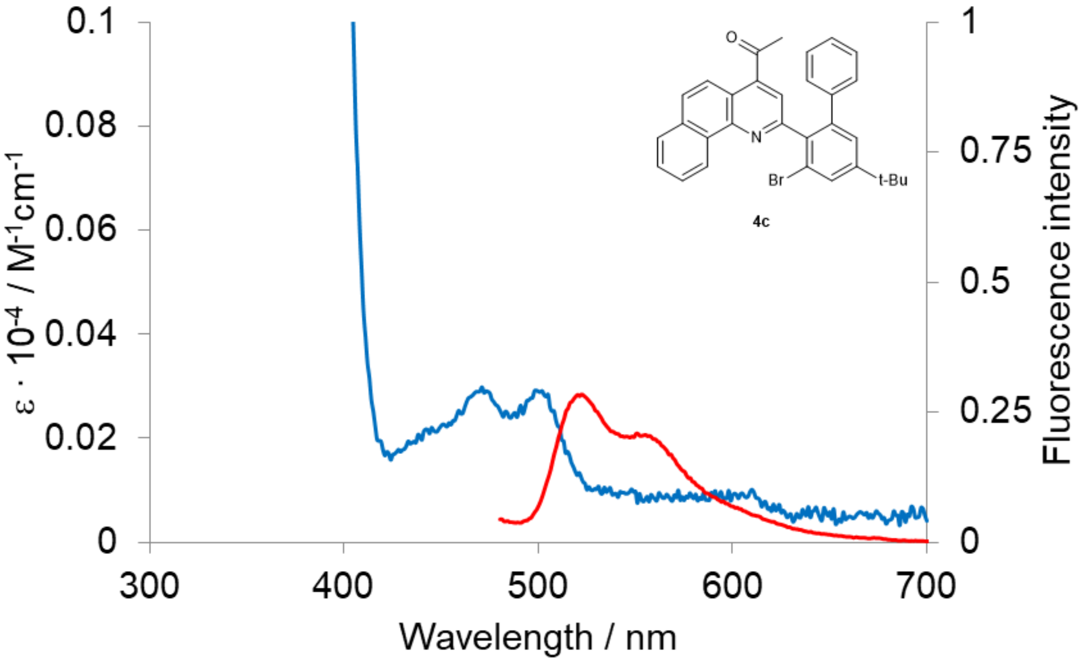
0.1
0.08
0.06
0.04
0.02
0
ε · 10-4 / M-1cm-1
1
0.75
0.5
0.25
0
Fluorescence intensity
300
400
500
600
700
Wavelength / nm
O
N
Br
t-Bu
4c

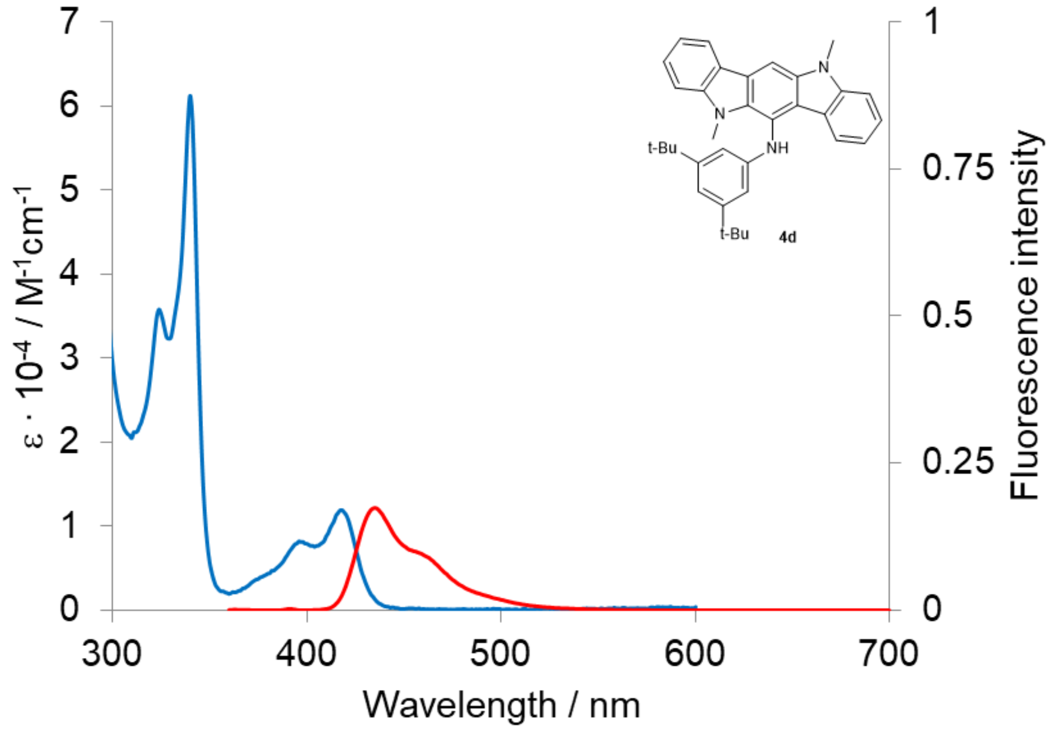

7
6
5
4
3
2
1
0
300
400
500
600
700
Wavelength / nm
ε · 10-4 / M-1cm-1
1
0.75
0.5
0.25
0
Fluorescence intensity
N
N
NH
t-Bu
t-Bu
4d

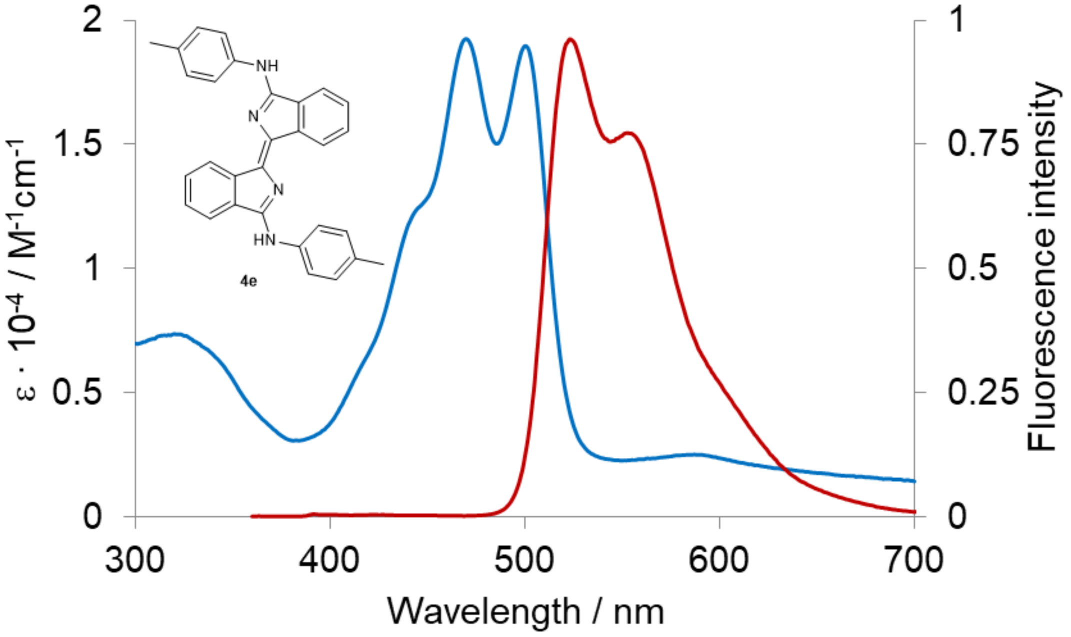

2
1.5
1
0.5
0
300
400
500
600
700
Wavelength / nm
ε · 10-4 / M-1cm-1
1
0.75
0.5
0.25
0
Fluorescence intensity
NH
N
N
HN
4e

Section S4. Supplementary References

S1. Hu, W.-J.; Liu, L.-Q.; Ma, M.-L.; Zhao, X.-L.; Liu, Y. A.; Mi, X.-Q.; Jiang, B.; Wen, K. M-Terphenyl-3,3″-Dioxo-Derived Oxacalixaromatics: Synthesis, Structure, and Solvent Encapsulation in the Solid State. *Tetrahedron* **2013**, *69* (19), 3934–3941.

S2. Chiba, Y.; Tanabe, T.; Koyama, S.; Yamanaka, S.; Takaishi, S.; Sakamoto, R.; Iguchi, H. Structure and Physical Properties of Alternately Stacked Donor-Acceptor Complexes with 5,11-Dimethyl-5,11-Dihydroindolo[3,2-*b*]Carbazole. *Chem. Lett.* **2023**, *52* (6), 488–491.

S3. Bruker, 2004, APEX2 and SAINT. Bruker AXS Inc., Madison, Wisconsin, USA.

S4. Sheldrick, G. M. A Short History of SHELX. *Acta Crystallogr. A* **2008**, *64* (Pt 1), 112–122.

S5. Sheldrick, G. M. Crystal Structure Refinement with SHELXL. *Acta Crystallogr. C Struct. Chem.* **2015**, *71* (Pt 1), 3–8.

S6. Bruker, 2008, SADABS. Bruker AXS Inc., Madison, Wisconsin, USA.

S7. G. M. Sheldrick, SHELXL-2014. Program for the Refinement of Crystal Structures from Diffraction Data, University of Göttingen, Germany (2014).
